# Factors associated with Nugent-bacterial vaginosis in pregnancy and postpartum among women in rural northwestern Bangladesh

**DOI:** 10.1371/journal.pgph.0004768

**Published:** 2025-06-13

**Authors:** Lena Kan, Susan Tuddenham, Parul Christian, Allan Massie, Alain B. Labrique, Lee Wu, Hasmot Ali, Mahbubur Rashid, Ethan Gough, Subhra Chakraborty, Michael T. France, Jacques Ravel, Keith P. West Jr, Daniel J. Erchick

**Affiliations:** 1 Department of International Health, Johns Hopkins Bloomberg School of Public Health, Baltimore, Maryland, United States of America; 2 Johns Hopkins University School of Medicine, Baltimore, Maryland, United States of America; 3 NYU Grossman School of Medicine Langone Health, New York, United States of America; 4 World Health Organization, Geneva, Switzerland; 5 JiVitA Maternal and Child Health Research Project, Rangpur, Bangladesh; 6 University of Maryland School of Medicine, Baltimore, Maryland, United States of America; PLOS: Public Library of Science, UNITED STATES OF AMERICA

## Abstract

Community-based longitudinal data on factors linked to bacterial vaginosis (BV) during and after pregnancy in Bangladesh are limited. Using data from a rural randomized trial of vitamin A and β-carotene supplementation, we examined factors associated with Nugent-score–assessed BV. Self-collected vaginal swabs from 1,812 participants were obtained in early pregnancy, late pregnancy, and 3 months postpartum for Nugent scoring. We analyzed associations between participant factors and Nugent-BV (scores 7–10 vs. 0–6; 4–10 vs. 0–3) at each time point. Bivariate associations were tested using chi-square and t-tests, and multivariable log-binomial regression was used to estimate adjusted prevalence ratios with 95% confidence intervals. In early pregnancy, consistent soap use during bathing (vs. never/sometimes) was associated with a decreased risk of Nugent-BV 7–10 (adjusted prevalence ratio (aPR): 0.64, 95% CI: 0.43, 0.96). In late pregnancy, Hindu religion (vs. Muslim) (aPR: 2.68, 95% CI: 1.52, 4.72) and higher gestational age (aPR: 1.18, 95% CI: 1.04, 1.35) were associated with increased risk of Nugent-BV 7–10 and 4–10. Furthermore, maternal underweight (BMI < 18.5 kg/m² vs. ≥ 18.5) (aPR: 0.62, 95% CI: 0.44, 0.87) and having ≥1 antenatal care visit (vs. none) (aPR: 0.59, 95% CI: 0.38, 0.91) were associated with reduced risk of Nugent-BV 4–10. Among multiparous individuals, a longer pregnancy interval of ≥18 months (vs. < 18 months) was protective against Nugent-BV 7–10 (aPR: 0.34, 95% CI: 0.14, 0.81). At 3-months postpartum, vitamin A supplementation (vs. placebo) was associated with a decreased risk of Nugent-BV 7–10, consistent with prior trial findings. Our findings indicate that Nugent-BV during pregnancy and postpartum is linked to modifiable factors, including hygiene, nutrition, birth spacing, and healthcare access. Rigorous randomized trials are needed to evaluate their ability to reduce BV, promote long-term vaginal health, and lower the risk of adverse pregnancy outcomes.

## Introduction

Bacterial vaginosis (BV) is a common condition, affecting nearly 30% of women worldwide, which can cause vaginal odor and discharge when symptomatic [1]. BV is characterized by a disruption of the bacterial communities colonizing the vagina (i.e., “vaginal microbiota”) and can be diagnosed in several ways [2], including with the Nugent score [3], which utilizes Gram-stained vaginal smears to characterize bacterial morphotypes present in the vagina. A Nugent score of 0–3 characterizes a vaginal bacterial community that is comprised of a high relative abundance of lactobacilli, while a score of 7–10 is diagnostic of “Nugent-BV” and represents a vaginal community lacking lactobacilli. Notably, Nugent-BV detected during pregnancy, whether symptomatic or asymptomatic, has been associated with subsequent preterm birth and other adverse pregnancy outcomes [4,5]. A Nugent score of 4–6 is considered “intermediate” and may also present some increased risk for adverse pregnancy outcomes [3]. Some researchers hypothesize that Nugent-BV postpartum could predispose women to adverse pregnancy outcomes in subsequent pregnancies, particularly when there is a short birth interval [5].

Few population-based estimates of Nugent-BV in pregnancy or postpartum are available from Bangladesh or South Asia more broadly, and little data is available on the associations between specific factors and risk of Nugent-BV [6] in this region. In other settings, lower education or income levels, younger age at first intercourse, frequency of intercourse, douching, smoking, increased number of sexual partners, sexually transmitted infection history, use of alternatives to water for intravaginal washing, and miscarriage or abortion history have been associated with Nugent-BV in pregnancy [7–16]. In rural Bangladesh, factors associated with risk of Nugent-BV may vary due to important differences in demographic characteristics, socioeconomic status, pregnancy history, nutrition, and sexual and hygiene behaviors. Novel biotherapeutics to treat Nugent-BV are under development in the U.S [17], and there is interest in whether, in the long term, such therapeutics could be used to prevent preterm birth and other adverse pregnancy outcomes in pregnant women. Context-specific data on factors associated with Nugent-BV are needed to support the design, testing, and targeting of new interventions to treat Nugent-BV and reduce risk for preterm birth and other outcomes, particularly in settings with high rates of these conditions, such as Bangladesh, where 19% of live births are preterm [18], and the newborn mortality rate is 16 deaths per 1,000 live births [19].

A previous large, community-based randomized controlled trial of vitamin A and β-carotene supplementation in pregnancy and postpartum [20–22], conducted in rural northwestern Bangladesh, found that weekly vitamin A supplementation reduced the prevalence (OR: 0.71; 95% CI: 0.52, 0.98) and incidence (RR: 0.58; 95% CI: 0.41, 0.81) of Nugent-BV 7–10 at three months postpartum compared to placebo, and both vitamin A and β-carotene reduced the prevalence and incidence of BV at either time point by 30–40% compared to placebo [20]. Utilizing data from this trial, controlling for supplementation group, we explored factors associated with Nugent-BV in early pregnancy, late pregnancy, and 3-months postpartum. The parent trial is registered at clinicaltrials.gov as NCT00198822 in the Am J Clin Nutr 2011; 94: 1643–9.

## Methods

### Study population and design

This study is a secondary analysis of data collected through JiVitA-1, a cluster-randomized, double-blinded, placebo-controlled trial of maternal vitamin A and β-carotene supplementation in pregnancy and postpartum conducted from 2001 to 2007 in the rural northwestern districts of Gaibandha and Rangpur in Bangladesh [23]. Over 60,000 women with incident pregnancies in an area divided into 596 communities (i.e., “sectors”) were enrolled into the trial. A pregnancy surveillance system identified pregnancies early in gestation and enrolled consenting participants to be dosed weekly with a micronutrient supplement (vitamin A: 7,000 lg retinol equivalents, or β-carotene: 42 mg, or placebo) in pregnancy and until 12 weeks postpartum [23]. The recruitment period for this study was between 18/09/2002 – 26/12/2006. A sub-study was introduced in August 2003 and nested within the JiVitA-1 trial to ascertain the burden of Nugent-BV and response to weekly vitamin A or β-carotene supplementation [20,23].

The BV sub-study, which was utilized for our analysis, enrolled 2,130 pregnant women in a sub-study area of 32 sectors in Jamalpur Union, Gaibandha District of the JiVitA-1 study area. Women identified as pregnant were enrolled and received household visits in early pregnancy, late pregnancy, and at 3-months postpartum [20]. Interviewers collected data on all trial participants, including demographic factors, socioeconomic status, pregnancy history, antenatal care utilization, and diet and nutrition. Within the sub-study, interviewers administered additional questionnaires on personal hygiene, reproductive symptoms, and sexual behaviors. Sub-study participants provided self-administered vaginal swabs for Nugent scoring and diagnosis of BV at the three time points. Vaginal swabs were collected at each time point regardless of whether swabs had been collected at previous visits to maximize the number of swabs collected at each cross-section.

Ethical approval for all procedures for the parent study was obtained from the Institutional Review Board of the Johns Hopkins Bloomberg School of Public Health and from the Bangladesh Medical Research Council. All participants provided oral informed consent. JiVitA data collectors visited participants in their homes to obtain and document oral consent for study participation. All consent procedures were approved by the Johns Hopkins Bloomberg School of Public Health Institutional Review Board and by the Bangladesh Medical Research Council (BMRC).

### Nugent-BV and participant factor definitions

At each BV sub-study visit, vaginal swabs (polyester-tipped sterile applicator swab, Puritan Medical Products, Guildford, ME) were self-collected by each participant, and questions were asked about vaginal symptoms. The swabs were then smeared by a data collector onto a glass microscope slide for Gram staining. Each slide underwent Nugent scoring by a study technician using an electric binocular microscope. The technician assigned a summed numerical score from 0-10 based on morphology and Gram’s stain of vaginal bacteria, including Gram positive rods *Lactobacillus* (decreasing score from 4-0), Gram variable cocci, e.g., *Gardnerella vaginalis* (increasing score from 0-4), and Gram variable curved rods, e.g., *Mobiluncus* (increasing score from 0-2) [3]. The median Nugent of ten fields for each sample was used to define Nugent-BV and categorized as either scores 7–10 or 4–10, intermediate Nugent-BV 4–6, and no Nugent-BV score 0–3. During the trial, symptomatic women with Nugent-BV 7–10 were offered treatment with a 7-day regimen of metronidazole (250 mg, by mouth 3 times a day) [20], according to the Bangladesh national guidelines at the time of the study.

Nugent-BV positive was defined as a Nugent score 7–10 or 4–10. Prevalence rates in early and late pregnancy, and at 3-months postpartum were calculated. Nugent-BV incidence was calculated with the numerator defined as new Nugent-BV cases among women without Nugent-BV at the previous assessment and the denominator as all women with a Nugent-BV assessment at both time points [20]. Participant characteristics and risk factor data for this analysis covered several domains, including demographic, socioeconomic, sexual behaviors, nutrition, personal and menstrual hygiene, and environmental. From the trial baseline visit, key variables used in this analysis included participant age, education, literacy, living standard (wealth) index (LSI), religion, anthropometry (body mass index (BMI), mid-upper arm circumference (MUAC)), marriage and pregnancy history, and betelnut, alcohol, and tobacco consumption. Variables measured at each vaginal sample collection time point included sexual activity, bathing practices, exposure to different water sources, and antibiotic treatment for BV. Variables only measured at the early pregnancy visit included use of soap when bathing, personal hygiene, and menstrual hygiene. The number of antenatal care visits for each participant was assessed by recall at the late pregnancy visit. Variables only measured at the 3-months postpartum visit included timing of resumed menstruation, if any, and family planning methods use. Wealth was assessed using a household living standard index (LSI) developed through a principal components analysis of the complete study data set [24]. Pregnancy outcomes were collected at a maternal and infant visit after delivery. Gestational age at end of pregnancy was calculated as the difference in days between the pregnancy end date and the last menstrual period (LMP) date as recalled by the mother at enrollment early in pregnancy, and gestational age at vaginal sample collection was calculated as the difference in days between the date of sample collection visit and the LMP date. In this study, our outcome is Nugent-BV at each study visit. In this analysis, we did not consider the effect of Nugent-BV in pregnancy on incidence of pregnancy outcomes or duration of pregnancy, which are a focus of a separate investigation. Rather, we focused on associations between demographic, behavioral, and clinical factors and Nugent-BV.

### Statistical analysis

Analyses were conducted separately in early pregnancy, late pregnancy, and 3-months postpartum. We calculated the prevalence and incidence of Nugent BV score 7–10, 4–10, and 4 6 for each visit. We assessed bivariate associations between each factor and Nugent-BV 7–10 (ref: 0–6), 4–10 (ref: 0–3), and 4–6 (ref: 0–3) using chi-squared tests for categorical variables and t-tests for continuous variables. Factors associated with Nugent-BV in bivariate analyses (p < 0.10) and those identified in previous literature as associated were selected for inclusion in final multivariable regression models. At each time point, we fitted separate regression models for each Nugent-BV exposure definition, 7–10 (ref: 0–6), 4–10 (ref: 0–3), and 4–6 (ref: 0–3). We used multivariable log binomial regression models to estimate adjusted prevalence ratios (aPR) of BV and associated 95% confidence intervals. All models used generalized estimating equations (GEE), assuming an exchangeable correlation structure, to account for the trial's cluster randomized study design. All regression models were adjusted for the JiVitA-1 trial intervention group (vitamin A supplementation, β-carotene supplementation, or placebo). To account for the increased risk of type I error due to multiple comparisons across the three time points, we adjusted the p-value for common variables in the multivariable regression models using the Benjamini-Hochberg procedure, which is a procedure to help control the rate of false positives and maximize statistical power [25,26]. All statistical analyses were conducted using STATA version 16.1 and the data were accessed on May 5th, 2024, for research purposes.

### Sensitivity analyses

Sensitivity analyses were conducted to evaluate additional factors associated with Nugent-BV 7–10 or 4–10 in bivariate analyses that were not included in final models for various reasons, including because they were very low prevalence or were not associated with Nugent-BV in bivariate analyses but were of interest for specific hypotheses. Importantly, the number of women who were symptomatic with Nugent-BV 7–10 and received treatment with oral metronidazole during the study was small; therefore, this factor was not included in final models but instead was explored in sensitivity analyses at late pregnancy and postpartum time points.

#### Ethics statement.

This study is a secondary analysis to describe BV risk factors and assess associations with maternal symptoms and adverse pregnancy outcomes using population-based data from rural Bangladesh. No authors had access to information that could identify individual participants during or after data collection.

#### Inclusivity in global research.

Additional information regarding the ethical, cultural, and scientific considerations specific to inclusivity in global research is included in the Supporting Information (Inclusivity in Global Research Questionnaire).

## Results

### Enrollment and data completeness

Among the n = 2,130 pregnant women that were enrolled in the sub-study, n = 1,812 (85.1%) provided at least one vaginal swab that yielded a Nugent score across the three time points for this analysis (S1 Fig). Nugent score data was available for n = 1,462 women in early pregnancy, n = 1,079 women in late pregnancy, and n = 1,445 women postpartum. Only 911 participants had Nugent score data in both early and late pregnancy and 727 participants had Nugent score data at all three time points (early pregnancy, late pregnancy, and 3-months postpartum).

### Prevalence and incidence of Nugent-BV

In early pregnancy, late pregnancy, and 3-months postpartum, the prevalence of Nugent-BV 7–10 was 7.6%, 6.5%, and 10.4%, Nugent-BV 4–10 was 11.8%, 12.9%, and 19.1%, and Nugent-BV 4–6 was 4.6%, 6.8%, and 9.7% (Table 1). Across all three time points, a total of 15.5%, 27.1%, and 13.4% of participants had Nugent-BV 7–10, 4–10, and 4–6 at least once, respectively.

**Table 1:** Prevalence and incidence of Nugent-BV 7-10, Nugent-BV 4-10, and Nugent-BV 4-6 in early pregnancy, late pregnancy, and 3-months postpartum.

	Nugent-BV 7–10		Nugent-BV 4–10		Nugent-BV 4–6	
	N	n(%)	N	n(%)	N	n(%)
**Prevalence** ^1^						
Overall	1,812	281 (15.5)	1,812	491 (27.1)	1,812	243 (13.4)
Pregnancy	1,630	165 (10.1)	1,630	279 (17.1)	1,548	124 (8.0)
Early pregnancy (first trimester)	1,462	111 (7.6)	1,462	173 (11.8)	1,351	62 (4.6)
Late Pregnancy (third trimester)	1,079	70 (6.5)	1,079	139 (12.9)	1,009	**69 (6.8)**
3-months postpartum	1,445	**150 (10.4)**	1,445	**276 (19.1)**	1,295	**126 (9.7)**
**Incidence** ^2^						
Late pregnancy	843	31 (3.7)	812	63 (7.8)	783	34 (4.3)
Postpartum	830	**72 (8.7)**	771	**118 (15.3)**	711	**58 (8.2)**

^1^Comparisons were late pregnancy to early pregnancy and 3-months postpartum to late pregnancy.

^2^Comparison was 3-months postpartum to late pregnancy.

Bold: p < 0.05.

The prevalence of Nugent-BV 4–6 was higher in late pregnancy than early pregnancy (p < 0.05), but the prevalences of Nugent-BV 7–10 and 4–10 were not significantly different between late and early pregnancy. The prevalences of Nugent-BV 7–10, 4–10, and 4–6 were significantly higher at 3-months postpartum than in early or late pregnancy, individually (p < 0.05, Table 1). The incidences of Nugent-BV 7–10, 4–10, and 4–6 were observed to be higher at 3-months postpartum compared to late pregnancy (p < 0.05, Table 1).

**Fig 1:**
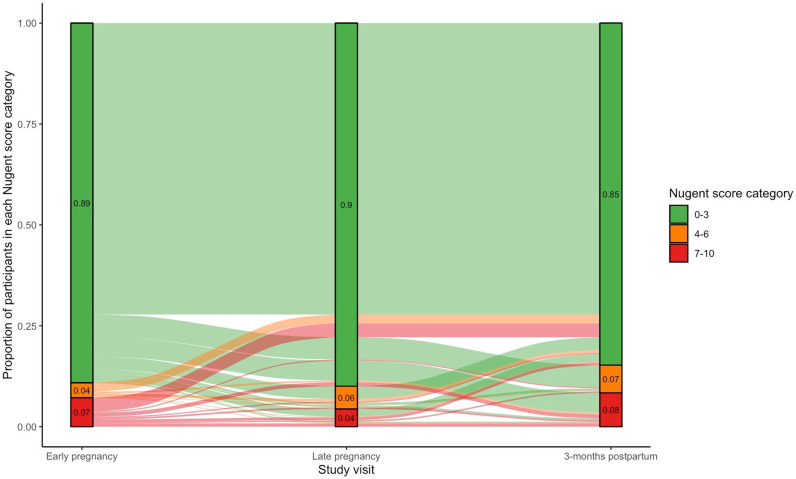
Nugent score transitions across early pregnancy, late pregnancy, and 3-months postpartum.

Among pregnant women with data at both pregnancy time points, the majority (n = 749, 92.2%) with Nugent 0–3 in early pregnancy maintained their status into late pregnancy. Few women with Nugent 0–3 in early pregnancy transitioned to Nugent-BV 4–6 (n = 34, 4.2%) or Nugent-BV 7–10 (n = 29, 3.6%) by late pregnancy. Of women with Nugent-BV 4–6 in early pregnancy, 22.6% (n = 7) maintained Nugent-BV 4–6, 71.0% (n = 22) transitioned to Nugent 0–3, and 6.5% (n = 2) transitioned to Nugent-BV 7–10 by late pregnancy. Of women with Nugent-BV 7–10 in early pregnancy, 23.5% (n = 16) maintained Nugent-BV 7–10, 11.8% (n = 8) transitioned to Nugent-BV 4–6, and 64.7% (n = 42) transitioned to Nugent 0–3 in late pregnancy. Among women with a Nugent score across all three time-points, 72.2% (n = 525) consistently reported Nugent 0–3, 13.5% (n = 98) transitioned from Nugent 0–3 in either early or late pregnancy to Nugent-BV 4–6 or Nugent-BV 7–10 in postpartum (Fig 1 and S1 Table).

During the study, n = 52 (2.9%), n = 27 (1.5%), and n = 53 (2.9%) women with symptomatic Nugent-BV 7–10 received study-administered treatment in early pregnancy, late pregnancy, and 3-months postpartum, respectively (Table A in S2 Table). The study protocol did not provide treatment for women with Nugent-BV 4–6 or those who were asymptomatic. Treatment was successful (i.e., no symptomatic BV at next visit) in 89.3% (n = 25) of women treated at early pregnancy and 72.2% (n = 13) of women treated at late pregnancy (Tables B and C in S2 Table). There was a statistically significantly (p < 0.05) lower percentage of BV at late pregnancy in those who were found to have BV and treated at early pregnancy as compared to those who had BV and were not treated. However, there was no statistically significant difference in the prevalence of BV based on treatment among those diagnosed in earlier time points when assessed at 3-months postpartum (Tables C and D in S2 Table).

### Bivariate associations between participant factors and Nugent-BV

The mean (SD) age of participants was 22.1 (6.4) years, 62.8% had some formal education, 49.8% of women were literate, and 93.7% were Muslim. Almost half of women (46.9%) were nulliparous. At trial enrollment, 43.2% were underweight (BMI <18.5 kg/m^2^). No women reported alcohol consumption and 9.6% reported tobacco use. Most (99.4%) reported having had sexual intercourse since the start of the pregnancy. Approximately three-quarters (75.7%) reported using cloth for menstruation prior to pregnancy, and only 2% reported using a disposable menstrual pad or other disposable method. Most reported changing the cloth every few hours (77.8%) or daily (22.2%), and nearly all washed the cloth with soap and water (98.3%). Over half (53.4%) did not use birth control before pregnancy, 35.9% used oral contraceptive pills, 7.1% used Depo-Provera injection, and 2.1% used condoms.

In early pregnancy, older age, lower education and literacy, less frequent use of soap when bathing, and using water only to wash menstrual cloth were associated in bivariate analyses with increased risk of Nugent-BV 7–10 or 4–10 (Table 2 and S3 Table). In late pregnancy, lower women's literacy and lower husband's education and literacy, higher gestational age at vaginal sample collection, lower household LSI, lower BMI (<18.5), shorter intrapartum period among those multiparous, receiving no antenatal care visits, and not receiving antibiotic treatment for symptomatic BV diagnosis in early pregnancy were associated with increased risk of Nugent-BV 7–10 or 4–10 (S4 and S5 Tables). At 3-months postpartum, several variables were associated with Nugent-BV 7–10 or 4–10, including older age, lower BMI (<18.5), lower MUAC, higher gestational age at pregnancy outcome (weeks), shorter intrapartum period among multiparous women, having a preterm birth, lower women's education and literacy, less frequent use of soap when bathing in early pregnancy, less frequent of washing the birth canal when bathing, non-use and non-reuse of cloth during menstruation prior to pregnancy, not using any form of family postpartum planning, and not receiving antibiotic treatment for symptomatic BV diagnosis in early or late pregnancy (S6 and S7 Tables).

**Table 2:** Characteristics of pregnant women by Nugent-BV 7-10 and 4-10 status in early pregnancy.

	Nugent-BV 0–6 (n = 1,351)	Nugent-BV 7–10 (n = 111)	p-value	Nugent-BV 0–3 (n = 1,289)	Nugent-BV 4–10 (n = 173)	p-value
**Clinical characteristics**						
**Age at pregnancy ascertainment (y), mean (SD)**	22.1 (6.4)	23.0 (7.3)	0.15	**22.0 (6.4)**	**23.3 (7.3)**	**0.02**
**Age (yrs)**			**0.09**			**0.01**
< 18	**364 (27.0%)**	**28 (25.2%)**		**350 (27.2%)**	**42 (24.3%)**	
18–29	**788 (58.4%)**	**58 (52.3%)**		**755 (58.6%)**	**91 (52.6%)**	
≥ 30	**198 (14.7%)**	**25 (22.5%)**		**183 (14.2%)**	**40 (23.1%)**	
**BMI (category)**			0.86			0.90
Underweight	573 (42.4%)	47 (42.3%)		544 (42.2%)	76 (43.9%)	
Normal weight	741 (54.9%)	62 (55.9%)		710 (55.1%)	93 (53.8%)	
Overweight or obese	36 (2.7%)	2 (1.8%)		34 (2.6%)	4 (2.3%)	
**BMI (category 2)**			0.98			0.67
Normal BMI	777 (57.6%)	64 (57.7%)		744 (57.8%)	97 (56.1%)	
Low BMI (<18.5)	573 (42.4%)	47 (42.3%)		544 (42.2%)	76 (43.9%)	
**Mid-upper arm circumference**			0.23			0.14
< 20	52 (4.0%)	8 (7.3%)		48 (3.8%)	12 (7.1%)	
≥ 20– < 23	633 (48.4%)	49 (44.5%)		603 (48.3%)	79 (47.0%)	
≥ 23	622 (47.6%)	53 (48.2%)		598 (47.9%)	77 (45.8%)	
**Parity**			0.41			0.14
0	658 (48.8%)	51 (45.9%)		631 (49.0%)	78 (45.3%)	
1–2	476 (35.3%)	37 (33.3%)		456 (35.4%)	57 (33.1%)	
3+	214 (15.9%)	23 (20.7%)		200 (15.5%)	37 (21.5%)	
**Gestational age at vaginal sample collection** ^1^	11.3 (4.8)	11.5 (4.8)	0.68	11.4 (4.9)	11.2 (4.5)	0.78
**Weeks since last pregnancy**	268.0 (149.1)	262.2 (109.3)	0.77	266.1 (144.8)	277.1 (156.4)	0.49
**Months since last pregnancy (category)**			0.32			0.96
< 18 months	46 (6.4%)	2 (3.2%)		42 (6.2%)	6 (6.1%)	
≥ 18 months	670 (93.6%)	60 (96.8%)		637 (93.8%)	93 (93.9%)	
**Age at first marriage (yrs)**			0.94			0.62
< 15	705 (57.9%)	59 (59.0%)		675 (57.9%)	89 (58.2%)	
15–18	340 (27.9%)	28 (28.0%)		322 (27.6%)	46 (30.1%)	
18+	173 (14.2%)	13 (13.0%)		168 (14.4%)	18 (11.8%)	
**Outcome of last pregnancy**			0.50			0.49
At least one live birth	608 (84.1%)	55 (87.3%)		581 (84.7%)	82 (82.0%)	
Stillbirth/miscarriage	115 (15.9%)	8 (12.7%)		105 (15.3%)	18 (18.0%)	
**SES characteristics**						
**Women’s education**			**0.03**			**0.02**
No schooling	**476 (35.3%)**	**53 (48.2%)**		**450 (34.9%)**	**79 (45.9%)**	
Class 1–7	**491 (36.4%)**	**32 (29.1%)**		**469 (36.4%)**	**54 (31.4%)**	
Class 8–14	**383 (28.4%)**	**25 (22.7%)**		**369 (28.6%)**	**39 (22.7%)**	
**Husband’s education**			0.59			0.22
No schooling	591 (46.5%)	53 (51.5%)		560 (46.1%)	84 (52.8%)	
Class 1–7	310 (24.4%)	24 (23.3%)		297 (24.4%)	37 (23.3%)	
Class 8–14	371 (29.2%)	26 (25.2%)		359 (29.5%)	38 (23.9%)	
**Living standard index**			0.59			0.20
Lowest	405 (30.0%)	37 (33.3%)		380 (29.5%)	62 (35.8%)	
Middle	456 (33.8%)	39 (35.1%)		438 (34.0%)	57 (32.9%)	
High	489 (36.2%)	35 (31.5%)		470 (36.5%)	54 (31.2%)	
**Women literacy**	**686 (50.8%)**	**47 (42.3%)**	**0.09**	**659 (51.2%)**	**74 (42.8%)**	**0.04**
**Husband literacy**	665 (49.5%)	47 (42.7%)	0.17	**639 (49.8%)**	**73 (42.4%)**	**0.07**
**Religion**			0.19			0.43
Muslim	1,270 (94.1%)	101 (91.0%)		1,211 (94.0%)	160 (92.5%)	
Hindu	80 (5.9%)	10 (9.0%)		77 (6.0%)	13 (7.5%)	
**Behavioral characteristics** ^2^						
**Use soap when bathing** ^3^			**0.002**			**0.005**
Never/Sometimes	**335 (25.8%)**	**43 (39.4%)**		**318 (25.7%)**	**60 (35.9%)**	
Always	**962 (74.2%)**	**66 (60.6%)**		**921 (74.3%)**	**107 (64.1%)**	
**Water source when bathing**			0.30			0.15
Not pond/river/lake	289 (22.3%)	29 (26.6%)		273 (22.0%)	45 (26.9%)	
**Wash vaginal area when bathing**			0.88			0.54
No	183 (14.2%)	16 (14.7%)		178 (14.4%)	21 (12.7%)	
Yes	1,109 (85.8%)	93 (85.3%)		1,057 (85.6%)	145 (87.3%)	
**Wash vaginal area frequency**			0.97			0.81
Occasionally	312 (28.2%)	26 (28.0%)		296 (28.0%)	42 (29.0%)	
Every time	796 (71.8%)	67 (72.0%)		760 (72.0%)	103 (71.0%)	
**Clean anal area after defecation**			0.35			0.14
Front to back	731 (57.1%)	62 (57.9%)		697 (56.9%)	96 (58.9%)	
Back to front	139 (10.9%)	7 (6.5%)		136 (11.1%)	10 (6.1%)	
Either way	411 (32.1%)	38 (35.5%)		392 (32.0%)	57 (35.0%)	
**Entered water up to hips in last 30 days**			0.37			0.76
Never up to hips	1,228 (94.7%)	101 (92.7%)		1,172 (94.6%)	157 (94.0%)	
Up to hips	69 (5.3%)	8 (7.3%)		67 (5.4%)	10 (6.0%)	
**Used menstrual cloth prior to pregnancy** ^4^			0.66			0.25
No	308 (23.8%)	28 (25.7%)		290 (23.5%)	46 (27.5%)	
Yes	986 (76.2%)	81 (74.3%)		946 (76.5%)	121 (72.5%)	
**Re-used menstrual cloth**			0.98			0.97
No	24 (2.4%)	2 (2.5%)		23 (2.4%)	3 (2.5%)	
Yes	962 (97.6%)	79 (97.5%)		923 (97.6%)	118 (97.5%)	
**Water source to wash menstrual cloth**			**0.035**			**0.008**
Water only	**5 (0.5%)**	**2 (2.5%)**		**4 (0.4%)**	**3 (2.5%)**	
Water and soap/alkali	**957 (99.5%)**	**77 (97.5%)**		**919 (99.6%)**	**115 (97.5%)**	
**Used a disposable menstrual pad or other disposable method**			0.94			0.90
No	1,270 (98.1%)	107 (98.2%)		1,213 (98.1%)	164 (98.2%)	
Yes	25 (1.9%)	2 (1.8%)		24 (1.9%)	3 (1.8%)	
**Family planning use prior to pregnancy**			0.65			0.14
No FP	695 (53.6%)	55 (50.5%)		663 (53.5%)	87 (52.1%)	
Oral pills	469 (36.2%)	38 (34.9%)		450 (36.3%)	57 (34.1%)	
IUD	7 (0.5%)	1 (0.9%)		5 (0.4%)	3 (1.8%)	
Norplant	6 (0.5%)	1 (0.9%)		6 (0.5%)	1 (0.6%)	
Depo-Provera injection	86 (6.6%)	12 (11.0%)		81 (6.5%)	17 (10.2%)	
Condoms	28 (2.2%)	2 (1.8%)		28 (2.3%)	2 (1.2%)	
Other	6 (0.5%)	0 (0.0%)		6 (0.5%)	0 (0.0%)	
**Self-reported BV treatment** ^5^			0.64			0.81
Oral tablet/syrup	107 (70.4%)	12 (85.7%)		105 (70.9%)	14 (77.8%)	
Intravaginal tablet	4 (2.6%)	0 (0.0%)		4 (2.7%)	0 (0.0%)	
Leaves or some herbal preparation	26 (17.1%)	1 (7.1%)		25 (16.9%)	2 (11.1%)	
Other	15 (9.9%)	1 (7.1%)		14 (9.5%)	2 (11.1%)	
**Chewed betelnut**			0.57			0.38
No	413 (31.9%)	38 (34.5%)		392 (31.7%)	59 (35.1%)	
Yes	881 (68.1%)	72 (65.5%)		844 (68.3%)	109 (64.9%)	

^1^n = 371/1,748 (21.2%) women had gestational age > 98 days at the early pregnancy visit.

^2^No women reported consuming alcohol; < 10% reported any tobacco use at enrollment; 99.4% of women reported having sexual intercourse since getting pregnant. However, frequency of sexual intercourse since the start of pregnancy was not associated with BV during early or late pregnancy, and frequency of sexual intercourse after pregnancy was not associated with BV at three months postpartum.

^3^n = 5 (0.35%) women reported never using soap when bathing in early pregnancy.

^4^Even though some variables related to using menstrual cloth prior to pregnancy were significantly associated with Nugent-BV 7–10 or Nugent-BV 4–10 in early pregnancy, they were excluded from sensitivity analyses and the final regression models given that ~98% reported re-using menstrual cloth in early pregnancy, and ~99% women reported using water and soap/alkali to wash menstrual cloth if they reused it. Furthermore, it is less clear how relevant these indicators may be, provided the data were collected in early pregnancy and menses did not occur during pregnancy.

^5^Type of BV treatment method was asked about in early pregnancy and refers to any recent treatment the woman received for abnormal vaginal discharge.

Bold: p < 0.1.

### Multivariable regression analyses

Of the participant factors included in the final multivariable regression models for early pregnancy, consistent use of soap when bathing (ref: never/sometimes using soap) was associated with decreased risk of Nugent-BV 7–10 (aPR: 0.64, 95% CI: 0.43, 0.96) (Table 3). Also in early pregnancy, maternal age ≥ 30 (ref: < 18) was associated with increased risk of Nugent-BV 4–10 (aPR: 2.00, 95% CI: 1.04, 3.86) prior to adjustment for multiple comparisons across time points, but not afterwards (p = 0.12) (Table 3 and S8 Table). In late pregnancy, Hindu religion (ref: Muslim religion) was associated with increased risk of Nugent-BV 7–10 (aPR: 2.68, 95% CI: 1.52, 4.72) and Nugent-BV 4–10 (aPR: 1.88, 95% CI: 1.28, 2.77) (Table 3). Similarly, in late pregnancy, higher gestational age (weeks since LMP) at sample collection was associated with increased risk of Nugent-BV 7–10 (aPR: 1.18, 95% CI: 1.04, 1.35) and Nugent-BV 4–10 (aPR: 1.17, 95% CI: 1.05, 1.30) (Table 3). Also, in late pregnancy, maternal low BMI (<18.5) at trial enrollment (i.e., early pregnancy) (aPR 0.62, 95% CI: 0.44, 0.87) and receiving ≥1 antenatal care (ANC) visits during pregnancy (ref: 0 visits) (aPR 0.59, 95% CI: 0.38, 0.91) were associated with decreased risk of Nugent-BV 4–10 (Table 3 and S8 Table). In late pregnancy, higher living standard index (ref: lowest living standard index) was associated with increased risk of Nugent-BV 4–10 (aPR: 0.55, 95% CI: 0.32, 0.94) prior to adjustment for multiple comparison across time points, but not afterwards (p = 0.09) (Table 3). We adjusted for the supplementation group in all study models, and our findings confirmed previously published results, specifically a reduced risk of postpartum Nugent-BV in the vitamin A group compared to the placebo group [20].

**Table 3:** Adjusted associations between Nugent-BV 7-10 and 4-10 and potential factors in early pregnancy, late pregnancy, and 3-months postpartum.

Variables	Early pregnancy (n = 1,351)		Late pregnancy (n = 970)		Postpartum (n = 1,428)	
	Nugent-BV 7–10	Nugent-BV 4–10	Nugent-BV 7–10	Nugent-BV 4–10	Nugent-BV 7–10	Nugent-BV 4–10
**Age**						
< 18	Ref	Ref	Ref	Ref	Ref	Ref
18–29	1.09 (0.60, 1.99)	1.19 (0.77, 1.84)	0.72 (0.38, 1.35)	0.80 (0.57, 1.11)	0.66 (0.42, 1.04)	0.87 (0.64, 1.18)
≥ 30	1.72 (0.79, 3.76)	**2.00 (1.04, 3.86)**	0.74 (0.18, 3.08)	0.85 (0.37, 1.97)	0.94 (0.50, 1.79)	1.14 (0.71, 1.83)
**BMI**						
Normal BMI	Ref	Ref	Ref	Ref	Ref	Ref
Low BMI (<18.5)	0.94 (0.71, 1.23)	0.97 (0.78, 1.21)	0.74 (0.45, 1.22)	0.62 (0.44, 0.87)*	1.16 (0.82, 1.63)	1.02 (0.98, 1.06)
**GA at vaginal sample collection (EP/LP)/ weeks since delivery (PP)** ^2^	1.00 (0.97, 1.04)	0.99 (0.96, 1.03)	1.18 (1.04, 1.35)*	1.17 (1.05, 1.30)*	0.98 (0.94, 1.03)	1.16 (0.89, 1.50)
**Wealth (LSI)** ^3^						
Lowest	Ref	Ref	Ref	Ref	Ref	Ref
Middle	1.05 (0.69, 1.62)	0.91 (0.68, 1.23)	0.84 (0.41, 1.69)	0.93 (0.62, 1.41)	0.96 (0.62, 1.47)	0.88 (0.62, 1.24)
High	1.05 (0.64, 1.73)	0.94 (0.65, 1.36)	0.51 (0.24, 1.08)	**0.55 (0.32, 0.94)**	0.74 (0.45, 1.21)	0.82 (0.56, 1.20)
**Maternal education** ^4^						
No education	Ref	Ref	Ref	Ref	Ref	Ref
Class 1–7	0.72 (0.42, 1.23)	0.81 (0.54, 1.23)	0.81 (0.44, 1.52)	0.88 (0.61, 1.26)	0.97 (0.65, 1.45)	1.02 (0.79, 1.32)
Class 8–14	0.72 (0.41, 1.25)	0.73 (0.46, 1.18)	1.10 (0.51, 2.39)	0.81 (0.47, 1.40)	0.72 (0.41, 1.25)	0.95 (0.66, 1.36)
**Religion**						
Muslim	Ref	Ref	Ref	Ref	Ref	Ref
Hindu	1.65 (0.86, 3.17)	1.32 (0.74, 2.36)	2.68 (1.52, 4.72)*	1.88 (1.28, 2.77)*	1.09 (0.61, 1.93)	0.80 (0.46, 1.39)
**Parity**						
0	Ref	Ref	Ref	Ref	Ref	Ref
1–2	0.80 (0.43, 1.48)	0.77 (0.55, 1.07)	1.04 (0.45, 2.42)	0.80 (0.52, 1.24)	1.01 (0.66, 1.53)	1.00 (0.74, 1.34)
3+	0.77 (0.39, 1.52)	0.80 (0.49, 1.30)	0.73 (0.20, 2.62)	0.72 (0.39, 1.33)	0.94 (0.56, 1.59)	1.14 (0.81, 1.58)
**Use soap when bathing**						
Never/Sometimes	Ref	Ref	–	–	–	–
Always	0.64 (0.43, 0.96)*	0.77 (0.59, 1.02)	–	–	–	–
**Antenatal care visits** ^5^						
0 visits	–	–	Ref	Ref	–	–
At least 1 visit	–	–	0.53 (0.26, 1.09)	0.59 (0.38, 0.91)*	–	–

^1^This table presents adjusted prevalence ratios and associated confidence intervals prior to multiple comparisons correction. Results marked with an asterisk indicate that the p-value for that test remained significant (p < 0.05) after Benjamini-Hochberg adjustment or that the p-value was significant and this test did not require multiple comparisons correction because this variable was only used in one final model. Bold tests without an asterisk indicate a test that was significant (p < 0.05) prior to Benjamini-Hochberg adjustment but not afterwards.

^2^GA: gestational age; EP: early pregnancy; LP: late pregnancy; PP: postpartum.

^3^LSI: living standard index.

^4^Husband’s education and women’s and husband’s literacy were excluded from multivariable models due to being correlated with women’s education.

^5^The antenatal care variable was only included in late pregnancy because we did not expect it to have any impact on early pregnancy or postpartum due to the timing of the visits (i.e., many women would not have started antenatal care by early pregnancy swab collection).

The regression models adjusted for trial supplementation group in each analysis.

### Sensitivity analyses

Among multiparous individuals, decreased time since the last pregnancy, defined as a short pregnancy interval of <18 months (ref: ≥ 18 months), was associated with Nugent-BV 7–10 (aPR: 0.34, 95% CI: 0.14, 0.81) in late pregnancy (Table B1 in S9 Table). Also in late pregnancy, those with untreated symptomatic BV (ref: no diagnosis of symptomatic BV) in early pregnancy were more likely to have Nugent-BV 7–10 (Table B2 in S9 Table). A diagnosis of symptomatic BV in early or late pregnancy, whether treated or untreated (ref: no diagnosis of symptomatic BV), was strongly associated with Nugent-BV 7–10 and 4–10 at 3-months postpartum (significant aPRs ranging from 1.93-3.33) (Table C1 in S9 Table). Additionally, consistent use of soap when bathing (ref: never/sometimes using soap) was associated with a decreased risk of Nugent BV 4–10 at 3-months postpartum (aPR: 0.71, 95% CI: 0.53, 0.93) (Table C2 in S9 Table).

In our sensitivity analysis, we looked at all pregnancy outcomes (term birth, preterm birth, miscarriage, stillbirth, or abortion) and assessed how they were associated with BV at 3 months postpartum. In the 3-months postpartum multivariable models, miscarriage (ref: term birth) was associated with decreased risk of Nugent-BV 4–10 (aPR 0.52, 95% CI: 0.32, 0.85) (and Nugent-BV 7–10, though not significant; Table C3 in S9 Table). Stillbirth and abortion outcomes were similarly protective for both Nugent-BV 7–10 and 4–10 status at 3-months postpartum, though not statistically significant (Table C3 in S9 Table). Additional variables that were selected for sensitivity analyses but not included in the final models are summarized in S9 and S10 Tables.

## Discussion

In a rural cohort in northwestern Bangladesh, several factors were associated with increased risk of Nugent-BV in pregnancy, including Hindu religion, advancing gestational age, and short pregnancy interval. Other factors were associated with decreased risk of Nugent-BV in pregnancy, including improved personal hygiene such as soap use, access to antenatal care visits in pregnancy, and maternal low BMI entering pregnancy. Maternal supplementation in pregnancy with vitamin A was associated with decreased risk of Nugent-BV at 3-months postpartum in this adjusted analysis as previously shown [20]. In a sensitivity analysis, miscarriage was associated with decreased risk of Nugent-BV at 3-months postpartum. Together, these results suggest that BV in pregnancy and postpartum may have multiple drivers, including modifiable factors such as hygiene practices, nutritional status, birth spacing, and access to care. Associated factors were often shared or showed similar trends across Nugent-BV 7–10 and 4–6, suggesting that Nugent-BV 4–10 may represent a spectrum of vaginal dysbiosis with similar predisposing factors. Future observational and interventional studies will be needed to further assess these factors and their relationship with BV.

Notably, factors which were associated with Nugent-BV 7–10 or 4–10 differed by pregnancy period, suggesting some discreet relationships may exist in early pregnancy, late pregnancy, and postpartum. Those with untreated Nugent-BV 7–10 in early pregnancy were more likely to have Nugent-BV 7–10 in late pregnancy than those who did not have Nugent-BV 7–10. In the postpartum period, those who had Nugent-BV 7–10 during pregnancy were more likely to have Nugent-BV 7–10 whether or not they were treated. This could reflect the poor long term efficacy of metronidazole; in other studies, nearly 60% of women have a recurrence of Nugent-BV 7–10 and nearly 70% have a recurrence of Nugent-BV 4–10 within 12 months after treatment [27]. It is also possible that the postpartum period is less favorable to *Lactobacilli*, and this may provide an opportunity for recurrence or relapse as BV-associated bacteria are able to thrive. In the literature, factors associated with Nugent-BV in pregnancy are less well characterized than in non-pregnant populations. Moreover, most previous analyses have been single time point cross-sectional studies [7–15,28], mainly conducted in early pregnancy, and not all have controlled for multiple factors in analysis. Factors associated with Nugent-BV during pregnancy have variously included lower education or income levels, younger age at first intercourse, frequency of intercourse, douching, smoking, increased number of sexual partners, STI history, use of alternatives to water for intravaginal washing, miscarriage or abortion history [7–16], however these have not been consistent or consistently evaluated across all studies.

In this generally undernourished population, our study found a significant reduction in postpartum Nugent-BV 7–10 among women in the vitamin A group compared to the placebo group and a non-significant reduction in postpartum Nugent-BV in the β-carotene group, aligning with our earlier reported trial findings [20]. Elsewhere, dietary intake and status of vitamin A have been associated with the composition of the vaginal microbiome among women of reproductive age, with some studies suggesting that low vitamin A intake may increase risk of BV [20,29–33], for which plausible pathways are emerging. Vitamin A deficiency may compromise epithelial integrity and the host immune response to infection [34–36]. Ensuring adequate Vitamin A status before and during pregnancy may be important for reducing the prevalence and incidence of BV during pregnancy and the postpartum period.

The other large study besides our own that has examined Nugent-BV 7–10 and 4–10 in pregnancy (n = 4,201) in Bangladesh [6] found that risk of Nugent-BV 7–10 and 4–10 was increased for those in lowest wealth quintile, with lower education of the woman or her husband, Hindu religion, and, in the case of Nugent-BV 4–10, with being in second versus first trimester in a multivariable analysis [6] – results that are broadly similar to those we obtained. The reason for associations with religion is not clear but could possibly be related to differences in male circumcision between the two groups; circumcision is nearly universal in Muslim populations [37] in Bangladesh, and likely much lower in Hindu populations, though published data are lacking. Circumcision has been associated with a lower risk of Nugent-BV among female partners in non-pregnant populations in the United States, Uganda, and in one small study in India [38,39]. The reason for lower risk of Nugent-BV 4–10 among underweight women at enrollment (low BMI) in our study is unclear. No such association was seen in the previous study from Bangladesh [17], though that study reported MUAC rather than BMI. The few previous studies examining associations between Nugent-BV and BMI in non-pregnant populations have had heterogenous results, with some finding increased risk of BV in those with a higher BMI and others finding increased risk in those who are underweight [40–42]. Other vaginal hygiene or sexual practices, such as douching or lack of condom use, have also been linked to Nugent-BV in non-pregnant populations [43,44]. However, our study did not identify any previously elucidated vaginal hygiene or sexual practices linked to Nugent-BV. Although sexual intercourse has been linked to increased risk of Nugent-BV in non-pregnant populations, almost all women reported sexual intercourse during pregnancy, so associations with this factor were not possible to assess. Menses, menstrual hygiene or contraception use have been linked to Nugent-BV in non-pregnant populations [45–50] but are not relevant during pregnancy.

In our cohort, prevalence of Nugent-BV 7–10, 4–10, and 4–6 were overall low. However, another notable finding from our study was that both the prevalence and incidence of all three states increased in the early postpartum compared to during pregnancy, suggesting women may be more vulnerable to Nugent-BV 7–10 in the postpartum period. We also found that among multiparous women, there was a decreased risk of Nugent-BV 7–10 with increased weeks since the last pregnancy, although sample size was limited. Few [51] studies have longitudinally assessed prevalence or incidence of Nugent-BV in the postpartum period as compared with pregnancy. However, our results are in line with several vaginal microbiome-based studies, which have suggested that vaginal *Lactobacilli* decrease and some other vaginal, including BV-associated, bacteria may increase in the postpartum period [52–58]. They are also in line with previous literature suggesting pregnant women are more likely than non-pregnant women to have a *Lactobacillus* dominated vaginal microbiota [59]. However, other studies have shown that *Lactobacillus* dominance increases with gestational age; it is not clear why our findings differed from these.

Short intrapartum period is known to increase risk of preterm birth [5,60,61]. Others have found an abrupt drop in *Lactobacilli* may occur in the vaginal microbiota post-partum [58]. Our findings are of interest in part because if women have an increased risk of Nugent-BV in the early postpartum period, it is possible that this is a factor that could put them at risk of poor obstetric outcomes in subsequent pregnancies, particularly when there is a short birth interval before the vaginal microbiota has a chance to recover [5]. If so, intervening on Nugent-BV postpartum could be a way to affect obstetric outcomes in subsequent pregnancies [58]. As shown previously, we found that weekly vitamin A supplementation reduced the risk of postpartum Nugent-BV, suggesting that micronutrient supplementation could be one such intervention. More research is needed to understand the time frame in which the vaginal microbiota in this population may recover to a *Lactobacillus*-dominated state after delivery.

There were some limitations to our study. First, some data was self-reported and may not always accurately reflect true behaviors, which could introduce bias and affect the reliability of study findings. However, the standardized and validated data collection procedures at the JiVitA research site, established in 2000, minimize measurement error and support the reliability of the data. No data was available on condom use or whether women or their partners had other concurrent partners, although rural Bangladesh is a highly monogamous context where intercourse occurs predominantly among married couples. Another limitation of the study is the absence of data on intrapartum antibiotic use, which could have a significant impact on the postpartum microbiota. Although our analyses controlled for study cluster and study arm and accounted for the cluster randomized design, it is possible the study and intervention could have influenced associations with Nugent-BV in late pregnancy and the postpartum period. Furthermore, no data was gathered in the second trimester. Not all factors were assessed across all stages of pregnancy. Finally, while Nugent scoring using Gram staining is a widely used method for diagnosing BV, it may not fully capture all aspects of vaginal microbiota dysbiosis. The strengths of our study lie in the large sample size and longitudinal sampling, which enabled us to examine factors across two trimesters in pregnancy and in the postpartum period.

Despite these factors, our study adds significantly to the literature exploring factors associated with Nugent-BV 7–10 and 4–10 across pregnancy and the postpartum period in Bangladesh. More research is needed to understand the relationships between various factors, Nugent-BV 7–10 and 4–10 throughout pregnancy as well as associations between Nugent-BV, higher risk vaginal microbiota, and adverse pregnancy outcomes in this population. Future research, including using new molecular techniques to longitudinally explore the impact of behavioral or clinical factors on BV and the vaginal microbiota in pregnancy is needed in this rural Bangladeshi population.

## Supporting information

S1 Fig Participant flowchart. (TIF)

S1 Table Nugent score distribution across early pregnancy, late pregnancy, and 3-months postpartum among all participants (n = 1,812). (DOC)

S2 Table Nugent-BV treatment variables in early pregnancy, late pregnancy, and 3-months postpartum. (DOC)

S3 Table Characteristics of pregnant women by Nugent-BV 4–6 in early pregnancy. (DOC)

S4 Table Characteristics of pregnant women by Nugent-BV 7–10 and Nugent-BV 4–10 in late pregnancy. (DOC)

S5 Table Characteristics of pregnant women by Nugent-BV 4–6 in late pregnancy. (DOC)

S6 Table Characteristics of pregnant women by Nugent-BV 7–10 and Nugent-BV 4–10 3 months postpartum. (DOC)

S7 Table Characteristics of pregnant women by Nugent-BV 4–6 3-months postpartum. (DOC)

S8 Table Adjusted associations between Nugent-BV 4–6 and potential factors in early and late pregnancy and 3-months postpartum. (DOC)

S9 Table Sensitivity analyses with additional potential factors associated with Nugent-BV 7–10, Nugent-BV 4–10, and Nugent-BV 4–6. (DOC)

S10 Table Summary of reasons why variables were excluded from the final regression models or sensitivity analyses. (DOC)
