# Supplementary material for: Factors associated with Nugent-bacterial vaginosis in pregnancy and postpartum among women in rural northwestern Bangladesh
Source: PLOS Glob Public Health. 2025 Jun 13;5(6):e0004768. doi: 10.1371/journal.pgph.0004768 (PMC12165353; doi:10.1371/journal.pgph.0004768)
Supplement: S1 Table — (DOC) [file pgph.0004768.s002.doc]

S1 Table. Nugent score distribution across early pregnancy, late pregnancy, and 3-months postpartum among all participants (n=1,812)

| **Nugent score (early)** | **Nugent score (late)** | **Nugent score (post-partum)** | **N** | **Proportion** |
| --- | --- | --- | --- | --- |
| 0-3 | 0-3 | 0-3 | 525 | 28.97% |
| 0-3 |  | 0-3 | 284 | 15.67% |
| 0-3 |  |  | 153 | 8.44% |
| 0-3 | 0-3 |  | 148 | 8.17% |
|  |  | 0-3 | 116 | 6.40% |
|  | 0-3 | 0-3 | 87 | 4.80% |
| 0-3 | 0-3 | 4-6 | 40 | 2.21% |
| 0-3 | 0-3 | 7-10 | 36 | 1.99% |
|  |  | 7-10 | 34 | 1.88% |
|  |  | 4-6 | 32 | 1.77% |
| 7-10 | 0-3 | 0-3 | 25 | 1.38% |
| 0-3 | 4-6 | 0-3 | 23 | 1.27% |
| 0-3 |  | 4-6 | 22 | 1.21% |
| 7-10 |  | 0-3 | 21 | 1.16% |
| 4-6 |  | 0-3 | 20 | 1.10% |
| 0-3 |  | 7-10 | 18 | 0.99% |
| 4-6 | 0-3 | 0-3 | 16 | 0.88% |
|  | 0-3 | 4-6 | 16 | 0.88% |
| 0-3 | 7-10 | 0-3 | 14 | 0.77% |
|  | 7-10 | 0-3 | 14 | 0.77% |
|  | 0-3 | 7-10 | 14 | 0.77% |
| 0-3 | 7-10 |  | 12 | 0.66% |
|  | 4-6 | 0-3 | 11 | 0.61% |
| 7-10 |  |  | 11 | 0.61% |
| 7-10 | 0-3 |  | 10 | 0.55% |
|  | 7-10 | 7-10 | 8 | 0.44% |
|  | 0-3 |  | 8 | 0.44% |
| 4-6 |  |  | 8 | 0.44% |
| 7-10 | 0-3 | 7-10 | 7 | 0.39% |
| 7-10 |  | 7-10 | 7 | 0.39% |
|  | 4-6 | 7-10 | 6 | 0.33% |
| 7-10 | 7-10 | 7-10 | 6 | 0.33% |
| 7-10 | 7-10 | 0-3 | 5 | 0.28% |
| 4-6 | 4-6 | 0-3 | 5 | 0.28% |
| 7-10 |  | 4-6 | 4 | 0.22% |
| 0-3 | 4-6 | 4-6 | 4 | 0.22% |
| 0-3 | 4-6 |  | 4 | 0.22% |
| 7-10 | 4-6 | 7-10 | 3 | 0.17% |
| 4-6 | 0-3 |  | 3 | 0.17% |
| 4-6 | 0-3 | 7-10 | 3 | 0.17% |
| 7-10 | 7-10 |  | 3 | 0.17% |
| 7-10 | 4-6 |  | 3 | 0.17% |
| 0-3 | 4-6 | 7-10 | 3 | 0.17% |
| 7-10 | 7-10 | 4-6 | 2 | 0.11% |
|  | 4-6 |  | 2 | 0.11% |
| 7-10 | 0-3 | 4-6 | 2 | 0.11% |
| 7-10 | 4-6 | 0-3 | 2 | 0.11% |
| 4-6 |  | 7-10 | 2 | 0.11% |
| 0-3 | 7-10 | 7-10 | 2 | 0.11% |
| 4-6 | 4-6 | 4-6 | 1 | 0.06% |
|  | 4-6 | 4-6 | 1 | 0.06% |
| 4-6 |  | 4-6 | 1 | 0.06% |
| 0-3 | 7-10 | 4-6 | 1 | 0.06% |
| 4-6 | 7-10 | 0-3 | 1 | 0.06% |
|  | 7-10 |  | 1 | 0.06% |
| 4-6 | 7-10 | 7-10 | 1 | 0.06% |
| 4-6 | 4-6 |  | 1 | 0.06% |
