# Supplementary material for: Factors associated with Nugent-bacterial vaginosis in pregnancy and postpartum among women in rural northwestern Bangladesh
Source: PLOS Glob Public Health. 2025 Jun 13;5(6):e0004768. doi: 10.1371/journal.pgph.0004768 (PMC12165353; doi:10.1371/journal.pgph.0004768)
Supplement: S2 Table — (DOC) [file pgph.0004768.s003.doc]

**S2 Tables: Nugent-BV treatment variables in early pregnancy, late pregnancy, and 3-months postpartum**

**S2 Table A**. Nugent-BV treatment among all women at early pregnancy, later pregnancy, and 3-months postpartum

| **Prevalence** | **Early pregnancy**  **(N,%)** | **Late pregnancy**  **(N,%)** | **Early or late pregnancy**  **(N,%)^1^** | **3-months postpartum**  **(N,%)** |
| --- | --- | --- | --- | --- |
| No treatment/no BV | 1,701 (93.87) | 1,742 (96.14) | 1,647 (90.89) | 1,662 (91.72) |
| BV/no treatment | 59 (3.26) | 43 (2.37) | 89 (4.91) | 97 (5.35) |
| BV/treatment | 52 (2.87) | 27 (1.49) | 76 (4.19) | 53 (2.92) |

**^1^**Nugent-BV treatment in early or late pregnancy was coded as: 0 = No treatment/not BV at both early and late pregnancy, 1 = BV/no treatment at either early or late pregnancy, 2= BV/treatment at either early or late pregnancy

**S2 Table B**. Nugent-BV treatment in early pregnancy and Nugent-BV in late pregnancy

| **BV treatment in early pregnancy** | **BV in late pregnancy^1^** | | **p-value**^2^ |
| --- | --- | --- | --- |
|  | BV (N,%) | No BV (N,%) |  |
| No treatment/no BV | 54 (5.34) | 957 (94.66) |  |
| BV/no treatment | 13 (32.50) | 27 (67.50) | **0.02** |
| BV/treatment | 3 (10.71) | 25 (89.29) |  |

^1^Chi^2^: p<0.001

^2^p-value was tested for BV in late pregnancy between treated vs. not treated women who had symptomatic BV in early pregnancy.

Bold: p<0.05

**S2 Table C. Nugent-BV treatment during late pregnancy and Nugent-BV 3-months** postpartum

| **BV treatment in late pregnancy** | **BV 3-months postpartum^1^** | | **p-value^2^** |
| --- | --- | --- | --- |
|  | BV (N,%) | No BV (N,%) |  |
| No treatment/no BV | 133 (9.56) | 1,258 (90.44) |  |
| BV/no treatment | 12 (33.33) | 24 (66.67) | 0.67 |
| BV/treatment | 5 (27.78) | 13 (72.22) |  |

^1^Chi^2^: p<0.001

^2^p-value was tested for BV postpartum between treated vs. not treated women who had symptomatic BV in late pregnancy.

**S2 Table D. Nugent-BV treatment during early or late pregnancy and Nugent-BV 3-months** postpartum

| **BV treatment in early or late pregnancy** | **BV 3-months postpartum^1^** | | **p-value**^2^ |
| --- | --- | --- | --- |
|  | BV (N,%) | No BV (N,%) |  |
| No treatment/no BV | 116 (8.79) | 1,204 (91.21) |  |
| BV/no treatment | 21 (29.17) | 51 (70.83) | 0.56 |
| BV/treatment | 13 (24.53) | 40 (75.47) |  |

^1^Chi^2^: p<0.001

^2^p-value was tested for BV postpartum between treated vs. not treated women who had symptomatic BV at either early or late pregnancy.
