# Supplementary material for: Factors associated with Nugent-bacterial vaginosis in pregnancy and postpartum among women in rural northwestern Bangladesh
Source: PLOS Glob Public Health. 2025 Jun 13;5(6):e0004768. doi: 10.1371/journal.pgph.0004768 (PMC12165353; doi:10.1371/journal.pgph.0004768)
Supplement: S4 Table — (DOC) [file pgph.0004768.s005.doc]

**S4 Table. Characteristics of pregnant women by Nugent-BV 7-10 and Nugent-BV 4-10 in late pregnancy**

|  | **Nugent 0-6 (n=1,009)** | **Nugent-BV 7-10 (n=70)** | **p-value** | **Nugent 0-3 (n=940)** | **Nugent-BV 4-10 (n=139)** | **p-value** |
| --- | --- | --- | --- | --- | --- | --- |
| **Treatment Arm** |  |  | 0.66 |  |  | 0.29 |
| Placebo | 311 (30.8%) | 25 (35.7%) |  | 285 (30.3%) | 51 (36.7%) |  |
| Vitamin A | 340 (33.7%) | 23 (32.9%) |  | 318 (33.8%) | 45 (32.4%) |  |
| Beta carotene | 358 (35.5%) | 22 (31.4%) |  | 337 (35.9%) | 43 (30.9%) |  |
| **Treatment adherence during pregnancy (0-100)** |  |  | 0.19 |  |  | 0.83 |
| <95 | 410 (40.6%) | 34 (48.6%) |  | 388 (41.3%) | 56 (40.3%) |  |
| ≥95 | 599 (59.4%) | 36 (51.4%) |  | 552 (58.7%) | 83 (59.7%) |  |
| **Clinical characteristics** | | | |  |  |  |
| **Age at pregnancy ascertainment (y), mean (SD)** | 21.1 (5.4) | 20.7 (5.2) | 0.52 | 21.1 (5.4) | 20.5 (5.4) | 0.24 |
| **Age (yrs)** |  |  | 0.19 |  |  | 0.14 |
| <18 | 284 (28.2%) | 24 (34.8%) |  | 259 (27.6%) | 49 (35.5%) |  |
| 18-29 | 638 (63.3%) | 39 (56.5%) |  | 600 (63.9%) | 77 (55.8%) |  |
| ≥30 | 86 (8.5%) | 6 (8.7%) |  | 80 (8.5%) | 12 (8.7%) |  |
| **BMI (category)** |  |  | 0.25 |  |  | 0.09 |
| Underweight | 414 (41.2%) | 25 (35.7%) |  | 393 (42.0%) | 46 (33.1%) |  |
| Normal weight | 567 (56.5%) | 45 (64.3%) |  | 521 (55.7%) | 91 (65.5%) |  |
| Overweight or obese | 23 (2.3%) | 0 (0.0%) |  | 21 (2.2%) | 2 (1.4%) |  |
| **BMI (category 2)** |  |  | 0.36 |  |  | **0.05** |
| Normal BMI | 590 (58.8%) | 45 (64.3%) |  | **542 (58.0%)** | **93 (66.9%)** |  |
| Low BMI (<18.5) | 414 (41.2%) | 25 (35.7%) |  | **393 (42.0%)** | **46 (33.1%)** |  |
| **Mid upper arm circumference** |  |  | 0.17 |  |  | 0.96 |
| <20 | 41 (4.1%) | 3 (4.3%) |  | 38 (4.0%) | 6 (4.3%) |  |
| ≥20-<23 | 478 (47.4%) | 41 (58.6%) |  | 451 (48.0%) | 68 (48.9%) |  |
| ≥23 | 490 (48.6%) | 26 (37.1%) |  | 451 (48.0%) | 65 (46.8%) |  |
| **Parity** |  |  | 0.84 |  |  | 0.52 |
| 0 | 526 (52.1%) | 39 (55.7%) |  | 486 (51.7%) | 79 (56.8%) |  |
| 1-2 | 389 (38.6%) | 25 (35.7%) |  | 366 (38.9%) | 48 (34.5%) |  |
| 3+ | 94 (9.3%) | 6 (8.6%) |  | 88 (9.4%) | 12 (8.6%) |  |
| **Gestational age at vaginal sample collection, mean (SD)^1^** | **32.4 (1.2)** | **32.8 (1.4)** | **0.02** | **32.4 (1.1)** | **32.8 (1.7)** | **<0.01** |
| **Weeks since last pregnancy** | **248.5 (126.6)** | **187.3 (119.3)** | **0.01** | 246.1 (125.7) | 233.6 (136.8) | 0.46 |
| **Months since last pregnancy (category)** |  |  | **0.01** |  |  | 0.39 |
| <18 months | **31 (6.2%)** | **6 (17.6%)** |  | 31 (6.6%) | 6 (9.5%) |  |
| ≥ 18 months | **468 (93.8%)** | **28 (82.4%)** |  | 439 (93.4%) | 57 (90.5%) |  |
| **Age at first marriage (yrs)** |  |  | 0.08 |  |  | 0.11 |
| <15 | 444 (54.5%) | 34 (70.8%) |  | 419 (54.4%) | 59 (64.1%) |  |
| 15-18 | 243 (29.9%) | 10 (20.8%) |  | 228 (29.6%) | 25 (27.2%) |  |
| 18+ | 127 (15.6%) | 4 (8.3%) |  | 123 (16.0%) | 8 (8.7%) |  |
| **Outcome of last pregnancy** |  |  | 0.41 |  |  | 0.76 |
| At least one live birth | 427 (84.7%) | 27 (79.4%) |  | 400 (84.2%) | 54 (85.7%) |  |
| Stillbirth/miscarriage | 77 (15.3%) | 7 (20.6%) |  | 75 (15.8%) | 9 (14.3%) |  |
| **SES characteristics** | | | |  |  |  |
| **Women’s education** |  |  | 0.55 |  |  | 0.07 |
| No schooling | 334 (33.1%) | 27 (38.6%) |  | 307 (32.7%) | 54 (38.8%) |  |
| Class 1-7 | 378 (37.5%) | 26 (37.1%) |  | 348 (37.0%) | 56 (40.3%) |  |
| Class 8-14 | 297 (29.4%) | 17 (24.3%) |  | 285 (30.3%) | 29 (20.9%) |  |
| **Husband’s education** |  |  | **0.04** |  |  | **0.02** |
| No schooling | **435 (45.7%)** | **35 (55.6%)** |  | **400 (45.1%)** | **70 (55.1%)** |  |
| Class 1-7 | **221 (23.2%)** | **18 (28.6%)** |  | **207 (23.3%)** | **32 (25.2%)** |  |
| Class 8-14 | **295 (31.0%)** | **10 (15.9%)** |  | **280 (31.6%)** | **25 (19.7%)** |  |
| **Living standard index** |  |  | 0.06 |  |  | **<0.01** |
| Lowest | 304 (30.1%) | 25 (35.7%) |  | **281 (29.9%)** | **48 (34.5%)** |  |
| Middle | 334 (33.1%) | 29 (41.4%) |  | **304 (32.3%)** | **59 (42.4%)** |  |
| High | 371 (36.8%) | 16 (22.9%) |  | **355 (37.8%)** | **32 (23.0%)** |  |
| **Women literacy** | 544 (53.9%) | 30 (42.9%) | 0.07 | **512 (54.5%)** | **62 (44.6%)** | **0.03** |
| **Husband literacy** | **509 (50.7%)** | **25 (36.2%)** | **0.02** | **480 (51.3%)** | **54 (39.1%)** | **0.01** |
| **Religion** |  |  | 0.12 |  |  | 0.38 |
| Muslim | 943 (93.5%) | 62 (88.6%) |  | 878 (93.4%) | 127 (91.4%) |  |
| Hindu | 66 (6.5%) | 8 (11.4%) |  | 62 (6.6%) | 12 (8.6%) |  |
| **Behavioral characteristics^2^** | | | |  |  |  |
| **Use soap when bathing^3^** |  |  | 0.15 |  |  | 0.51 |
| Never/Sometimes | 205 (23.6%) | 16 (32.7%) |  | 195 (23.8%) | 26 (26.8%) |  |
| Always | 664 (76.4%) | 33 (67.3%) |  | 626 (76.2%) | 71 (73.2%) |  |
| **Water source when bathing** |  |  | 0.13 |  |  | 0.12 |
| Not pond/river/lake | 238 (27.6%) | 10 (18.2%) |  | 227 (27.9%) | 21 (20.6%) |  |
| Pond/river/lake | 623 (72.4%) | 45 (81.8%) |  | 587 (72.1%) | 81 (79.4%) |  |
| **Wash vaginal area when bathing** |  |  | 0.055 |  |  | 0.89 |
| No | 125 (14.4%) | 12 (24.5%) |  | 122 (14.9%) | 15 (15.5%) |  |
| Yes | 741 (85.6%) | 37 (75.5%) |  | 696 (85.1%) | 82 (84.5%) |  |
| **Wash vaginal area**  **frequency** |  |  | 1.00 |  |  | 0.11 |
| Occasionally | 200 (27.0%) | 10 (27.0%) |  | 194 (27.9%) | 16 (19.5%) |  |
| Every time | 540 (73.0%) | 27 (73.0%) |  | 501 (72.1%) | 66 (80.5%) |  |
| **Clean anal area after defecation** |  |  | 0.48 |  |  | 0.49 |
| Front to back | 504 (58.6%) | 25 (51.0%) |  | 478 (58.9%) | 51 (52.6%) |  |
| Back to front | 93 (10.8%) | 5 (10.2%) |  | 86 (10.6%) | 12 (12.4%) |  |
| Either way | 263 (30.6%) | 19 (38.8%) |  | 248 (30.5%) | 34 (35.1%) |  |
| **Entered water up to hips in last 30 days^4^** |  |  | 0.58 |  |  | 0.33 |
| Never up to hips | 840 (97.6%) | 53 (96.4%) |  | 795 (97.7%) | 98 (96.1%) |  |
| Up to hips | 21 (2.4%) | 2 (3.6%) |  | 19 (2.3%) | 4 (3.9%) |  |
| **Used menstrual cloth prior to pregnancy^5^** |  |  | 0.18 |  |  | 0.56 |
| No | 194 (22.4%) | 7 (14.3%) |  | 182 (22.2%) | 19 (19.6%) |  |
| Yes | 674 (77.6%) | 42 (85.7%) |  | 638 (77.8%) | 78 (80.4%) |  |
| **Re-used menstrual**  **cloth** |  |  | 0.29 |  |  | 0.37 |
| No | 15 (2.2%) | 2 (4.8%) |  | 14 (2.2%) | 3 (3.8%) |  |
| Yes | 659 (97.8%) | 40 (95.2%) |  | 624 (97.8%) | 75 (96.2%) |  |
| **Water source to wash**  **menstrual cloth** |  |  | 0.096 |  |  | 0.36 |
| Water only | 3 (0.5%) | 1 (2.5%) |  | 3 (0.5%) | 1 (1.3%) |  |
| Water and soap/alkali | 656 (99.5%) | 39 (97.5%) |  | 621 (99.5%) | 74 (98.7%) |  |
| **Family planning use prior to pregnancy** |  |  | 0.92 |  |  | 0.41 |
| No FP | 490 (56.4%) | 32 (65.3%) |  | 460 (56.0%) | 62 (63.9%) |  |
| Oral pills | 294 (33.8%) | 14 (28.6%) |  | 282 (34.3%) | 26 (26.8%) |  |
| IUD | 3 (0.3%) | 0 (0.0%) |  | 2 (0.2%) | 1 (1.0%) |  |
| Norplant | 7 (0.8%) | 0 (0.0%) |  | 7 (0.9%) | 0 (0.0%) |  |
| Depo-Provera injection | 53 (6.1%) | 2 (4.1%) |  | 48 (5.8%) | 7 (7.2%) |  |
| Condoms | 20 (2.3%) | 1 (2.0%) |  | 20 (2.4%) | 1 (1.0%) |  |
| Other | 2 (0.2%) | 0 (0.0%) |  | 2 (0.2%) | 0 (0.0%) |  |
| **Self-reported BV treatment^6^** |  |  | 0.15 |  |  | 0.70 |
| Oral tablet/syrup | 61 (78.2%) | 1 (50.0%) |  | 57 (77.0%) | 5 (83.3%) |  |
| Intravaginal tablet | 5 (6.4%) | 0 (0.0%) |  | 5 (6.8%) | 0 (0.0%) |  |
| Salt water | 1 (1.3%) | 0 (0.0%) |  | 1 (1.4%) | 0 (0.0%) |  |
| Leaves or some herbal  preparation | 7 (9.0%) | 0 (0.0%) |  | 7 (9.5%) | 0 (0.0%) |  |
| Other | 4 (5.1%) | 1 (50.0%) |  | 4 (5.4%) | 1 (16.7%) |  |
| **Antenatal care visits** |  |  | **0.044** |  |  | **0.005** |
| 0 visits | **644 (68.4%)** | **47 (81.0%)** |  | **591 (67.6%)** | **100 (80.0%)** |  |
| At least 1 visit | **297 (31.6%)** | **11 (19.0%)** |  | **283 (32.4%)** | **25 (20.0%)** |  |
| **Antibiotic treatment for BV in early pregnancy^7^** |  |  | **<0.001** |  |  | **<0.001** |
| No treatment/no BV | **957 (94.8%)** | **54 (77.1%)** |  | **1,087 (93.0%)** | **233 (84.4%)** |  |
| BV/no treatment | **27 (2.7%)** | **13 (18.6%)** |  | **49 (4.2%)** | **23 (8.3%)** |  |
| BV/treatment | **25 (2.5%)** | **3 (4.3%)** |  | **33 (2.8%)** | **20 (7.2%)** |  |
| **Chewed betelnut** |  |  | 0.26 |  |  | 0.83 |
| No | 307 (30.7%) | 26 (37.1%) |  | 289 (31.0%) | 44 (31.9%) |  |
| Yes | 694 (69.3%) | 44 (62.9%) |  | 644 (69.0%) | 94 (68.1%) |  |

^1^n=1/1,335 (0.07%) women had gestational <196 days at the late pregnancy visit

^2^No women reported consuming alcohol; <10% reported any tobacco use at enrollment; 100% of women reported having sexual intercourse since getting pregnant in late pregnancy.

^3^n=5 (0.35%) women reported never using soap when bathing in early pregnancy

^4^Entering water up to hips for fishing, washing cows, or crossing water in last 30 days during late pregnancy was not associated with Nugent-BV 7-10 or Nugent-BV 4-10 in late pregnancy. Entering water for any other reason in last 30 days during late pregnancy was associated with Nugent-BV 7-10 and Nugent-BV 4-10 (n=2/12 and 3/12 women; p<0.1) in late pregnancy

^5^Even though some variables related to using menstrual cloth prior to pregnancy were significantly associated with Nugent-BV 7-10 or Nugent-BV 4-10 in early pregnancy, they were excluded from sensitivity analyses and the final regression models given that ~98% reported re-using menstrual cloth in early pregnancy, and ~99% women reported using water and soap/alkali to wash menstrual cloth if they reused it. Furthermore, it is less clear how relevant these indicators may be, provided the data were collected in early pregnancy and menses did not occur during pregnancy.

^6^Type of BV treatment method was asked in late pregnancy and refers to any recent treatment the woman received for abnormal vaginal discharge.

^7^Even though antibiotic treatment for BV during early pregnancy was significantly associated with Nugent-BV 7-10 or Nugent-BV 4-10 in late pregnancy, it was excluded from the final regression model because the proportion of women who had symptomatic BV and received treatment in early pregnancy was low.

Bold: p<0.1
