# Supplementary material for: Factors associated with Nugent-bacterial vaginosis in pregnancy and postpartum among women in rural northwestern Bangladesh
Source: PLOS Glob Public Health. 2025 Jun 13;5(6):e0004768. doi: 10.1371/journal.pgph.0004768 (PMC12165353; doi:10.1371/journal.pgph.0004768)
Supplement: S5 Table — (DOC) [file pgph.0004768.s006.doc]

S5 Table. Characteristics of pregnant women by Nugent-BV 4-6 in late pregnancy

|  | **Nugent 0-3**  **(n=940)** | **Nugent-BV 4-6**  **(n=69)** | **p-value** |
| --- | --- | --- | --- |
| **Treatment Arm** |  |  | 0.42 |
| Placebo | 285 (30.3%) | 26 (37.7%) |  |
| Vitamin A | 318 (33.8%) | 22 (31.9%) |  |
| Beta carotene | 337 (35.9%) | 21 (30.4%) |  |
| **Treatment adherence during pregnancy (0-100)** |  |  | 0.13 |
| <95 | 388 (41.3%) | 22 (31.9%) |  |
| ≥ 95 | 552 (58.7%) | 47 (68.1%) |  |
| **Clinical characteristics** | | | |
| **Age at pregnancy ascertainment (y), mean (SD)** | 21.1 (5.4) | 20.4 (5.6) | 0.31 |
| **Age (yrs)** |  |  | 0.29 |
| <18 | 259 (27.6%) | 25 (36.2%) |  |
| 18-29 | 600 (63.9%) | 38 (55.1%) |  |
| ≥30 | 80 (8.5%) | 6 (8.7%) |  |
| **BMI (category)** |  |  | 0.17 |
| Underweight | 393 (42.0%) | 21 (30.4%) |  |
| Normal weight | 521 (55.7%) | 46 (66.7%) |  |
| Overweight or obese | 21 (2.2%) | 2 (2.9%) |  |
| **BMI (category 2)** |  |  | 0.06 |
| Normal BMI | 542 (58.0%) | 48 (69.6%) |  |
| Low BMI (<18.5) | 393 (42.0%) | 21 (30.4%) |  |
| **Mid upper arm circumference** |  |  | 0.36 |
| <20 | 38 (4.0%) | 3 (4.3%) |  |
| ≥20-<23 | 451 (48.0%) | 27 (39.1%) |  |
| ≥23 | 451 (48.0%) | 39 (56.5%) |  |
| **Parity** |  |  | 0.59 |
| 0 | 486 (51.7%) | 40 (58.0%) |  |
| 1-2 | 366 (38.9%) | 23 (33.3%) |  |
| 3+ | 88 (9.4%) | 6 (8.7%) |  |
| **Gestational age at vaginal sample collection, mean (SD)^1^** | **32.4 (1.1)** | **32.8 (2.0)** | **0.01** |
| **Weeks since last pregnancy** | 236.3 (126.5) | 265.3 (133.6) | 0.25 |
| **Months since last pregnancy (category)** |  |  | 0.14 |
| <18 months | 34 (7.5%) | 0 (0.0%) |  |
| ≥ 18 months | 419 (92.5%) | 27 (100.0%) |  |
| **Age at first marriage (yrs)** |  |  | 0.45 |
| <15 | 419 (54.4%) | 25 (56.8%) |  |
| 15-18 | 228 (29.6%) | 15 (34.1%) |  |
| 18+ | 123 (16.0%) | 4 (9.1%) |  |
| **Outcome of last pregnancy** |  |  | 0.20 |
| At least one live birth | 400 (84.2%) | 27 (93.1%) |  |
| Stillbirth/miscarriage | 75 (15.8%) | 2 (6.9%) |  |
| **SES characteristics** | | | |
| **Women’s education** |  |  | 0.08 |
| No schooling | 307 (32.7%) | 27 (39.1%) |  |
| Class 1-7 | 348 (37.0%) | 30 (43.5%) |  |
| Class 8-14 | 285 (30.3%) | 12 (17.4%) |  |
| **Husband’s education** |  |  | 0.28 |
| No schooling | 400 (45.1%) | 35 (54.7%) |  |
| Class 1-7 | 207 (23.3%) | 14 (21.9%) |  |
| Class 8-14 | 280 (31.6%) | 15 (23.4%) |  |
| **Living standard index** |  |  | **0.04** |
| Lowest | **281 (29.9%)** | **23 (33.3%)** |  |
| Middle | **304 (32.3%)** | **30 (43.5%)** |  |
| High | **355 (37.8%)** | **16 (23.2%)** |  |
| **Women literacy** | 512 (54.5%) | 32 (46.4%) | 0.19 |
| **Husband literacy** | 480 (51.3%) | 29 (42.0%) | 0.14 |
| **Religion** |  |  | 0.80 |
| Muslim | 878 (93.4%) | 65 (94.2%) |  |
| Hindu | 62 (6.6%) | 4 (5.8%) |  |
| **Behavioral characteristics^2^** | | | |
| **Use soap when bathing^3^** |  |  | 0.64 |
| Never/Sometimes | 195 (23.8%) | 10 (20.8%) |  |
| Always | 626 (76.2%) | 38 (79.2%) |  |
| **Water source when bathing** |  |  | 0.50 |
| Not pond/river/lake | 227 (27.9%) | 11 (23.4%) |  |
| Pond/river/lake | 587 (72.1%) | 36 (76.6%) |  |
| **Wash vaginal area when bathing** |  |  | 0.097 |
| No | 122 (14.9%) | 3 (6.2%) |  |
| Yes | 696 (85.1%) | 45 (93.8%) |  |
| **Wash vaginal area**  **frequency** |  |  | **0.033** |
| Occasionally | **194 (27.9%)** | **6 (13.3%)** |  |
| Every time | **501 (72.1%)** | **39 (86.7%)** |  |
| **Clean anal area after defecation** |  |  | 0.66 |
| Front to back | 478 (58.9%) | 26 (54.2%) |  |
| Back to front | 86 (10.6%) | 7 (14.6%) |  |
| Either way | 248 (30.5%) | 15 (31.2%) |  |
| **Entered water up to hips in last 30 days^4^** |  |  | 0.41 |
| Never up to hips | 795 (97.7%) | 45 (95.7%) |  |
| Up to hips | 19 (2.3%) | 2 (4.3%) |  |
| **Used menstrual cloth prior to pregnancy** |  |  | 0.65 |
| No | 182 (22.2%) | 12 (25.0%) |  |
| Yes | 638 (77.8%) | 36 (75.0%) |  |
| **Re-used menstrual**  **cloth** |  |  | 0.82 |
| No | 14 (2.2%) | 1 (2.8%) |  |
| Yes | 624 (97.8%) | 35 (97.2%) |  |
| **Water source to wash**  **menstrual cloth** |  |  | 0.68 |
| Water only | 3 (0.5%) | 0 (0.0%) |  |
| Water and soap/alkali | 621 (99.5%) | 35 (100.0%) |  |
| **Family planning use prior to pregnancy** |  |  | 0.16 |
| No FP | 460 (56.0%) | 30 (62.5%) |  |
| Oral pills | 282 (34.3%) | 12 (25.0%) |  |
| IUD | 2 (0.2%) | 1 (2.1%) |  |
| Norplant | 7 (0.9%) | 0 (0.0%) |  |
| Depo-Provera injection | 48 (5.8%) | 5 (10.4%) |  |
| Condoms | 20 (2.4%) | 0 (0.0%) |  |
| Other | 2 (0.2%) | 0 (0.0%) |  |
| **Self-reported BV treatment^5^** |  |  | 0.88 |
| Oral tablet/syrup | 57 (77.0%) | 4 (100.0%) |  |
| Intravaginal tablet | 5 (6.8%) | 0 (0.0%) |  |
| Salt water | 1 (1.4%) | 0 (0.0%) |  |
| Leaves or some herbal  preparation | 7 (9.5%) | 0 (0.0%) |  |
| Other | 4 (5.4%) | 0 (0.0%) |  |
| **Antenatal care visits** |  |  | 0.051 |
| 0 visits | 591 (67.6%) | 53 (79.1%) |  |
| At least 1 visit | 283 (32.4%) | 14 (20.9%) |  |
| **Antibiotic treatment for BV in early pregnancy^6^** |  |  | **0.005** |
| No treatment/no BV | **896 (95.3%)** | **61 (88.4%)** |  |
| BV/no treatment | **21 (2.2%)** | **6 (8.7%)** |  |
| BV/treatment | **23 (2.4%)** | **2 (2.9%)** |  |
| **Chewed betelnut** |  |  | 0.44 |
| No | 289 (31.0%) | 18 (26.5%) |  |
| Yes | 644 (69.0%) | 50 (73.5%) |  |

^1^n=1/1,335 (0.07%) women had gestational age <196 days at the late pregnancy visit.

^2^No women reported consuming alcohol; <10% reported any tobacco use at enrollment; 100% of women reported having sexual intercourse since getting pregnant.

^3^n=5 (0.35%) women reported never using soap when bathing in early pregnancy.

^4^Entering water up to hips for fishing, washing cows, or for any other reason in the last 30 days during late pregnancy was not associated with Nugent-BV 4-6 in late pregnancy. Entering water to cross water in the last 30 days during late pregnancy was associated with Nugent-BV 4-6 (1/4 women; p<0.1).

^5^Type of BV treatment method was asked about in late pregnancy and refers to any recent treatment the woman received for abnormal vaginal discharge.

^6^Even though antibiotic treatment for BV during early pregnancy was significantly associated with Nugent-BV 4-6 in late pregnancy, it was excluded from the final regression model because the proportion of women who had symptomatic BV and received treatment in early pregnancy was low.

Bold: p<0.1
