# Supplementary material for: Factors associated with Nugent-bacterial vaginosis in pregnancy and postpartum among women in rural northwestern Bangladesh
Source: PLOS Glob Public Health. 2025 Jun 13;5(6):e0004768. doi: 10.1371/journal.pgph.0004768 (PMC12165353; doi:10.1371/journal.pgph.0004768)
Supplement: S6 Table — (DOC) [file pgph.0004768.s007.doc]

**S6 Table. Characteristics of pregnant women by Nugent-BV 7-10 and Nugent-BV 4-10** 3 months postpartum

|  | **Nugent 0-6 (n=1,295)** | **Nugent-BV 7-10 (n=150)** | **p-value** | **Nugent 0-3**  **(n=1,169)** | **Nugent-BV 4-10 (n=276)** | **p-value** |
| --- | --- | --- | --- | --- | --- | --- |
| **Treatment Arm** |  |  | 0.23 |  |  | 0.66 |
| Placebo | 397 (30.7%) | 56 (37.3%) |  | 362 (31.0%) | 91 (33.0%) |  |
| Vitamin A | 419 (32.4%) | 42 (28.0%) |  | 379 (32.4%) | 82 (29.7%) |  |
| Beta carotene | 479 (37.0%) | 52 (34.7%) |  | 428 (36.6%) | 103 (37.3%) |  |
| **Treatment adherence (0-100) during study** |  |  | 0.76 |  |  | 0.53 |
| <95 | 535 (41.3%) | 60 (40.0%) |  | 486 (41.6%) | 109 (39.5%) |  |
| ≥ 95 | 760 (58.7%) | 90 (60.0%) |  | 683 (58.4%) | 167 (60.5%) |  |
| **Clinical characteristics** | | | |  |  |  |
| **Age at pregnancy ascertainment (y), mean (SD)** | 22.2 (6.3) | 22.0 (7.2) | 0.78 | 22.0 (6.2) | 22.7 (7.1) | 0.13 |
| **Age (yrs)** |  |  | 0.06 |  |  | **0.05** |
| <18 | 327 (25.3%) | 48 (32.0%) |  | **300 (25.7%)** | **75 (27.2%)** |  |
| 18-29 | 786 (60.7%) | 76 (50.7%) |  | **712 (61.0%)** | **150 (54.3%)** |  |
| ≥30 | 181 (14.0%) | 26 (17.3%) |  | **156 (13.4%)** | **51 (18.5%)** |  |
| **BMI (category)** |  |  | 0.14 |  |  | 0.18 |
| Underweight | 535 (41.7%) | 71 (47.3%) |  | 477 (41.2%) | 129 (46.7%) |  |
| Normal weight | 710 (55.3%) | 78 (52.0%) |  | 646 (55.8%) | 142 (51.4%) |  |
| Overweight or obese | 38 (3.0%) | 1 (0.7%) |  | 34 (2.9%) | 5 (1.8%) |  |
| **BMI (category 2)** |  |  | 0.19 |  |  | **0.01** |
| Normal BMI | 748 (58.3%) | 79 (52.7%) |  | **680 (58.8%)** | **147 (53.3%)** |  |
| Low BMI (<18.5) | 535 (41.7%) | 71 (47.3%) |  | **477 (41.2%)** | **129 (46.7%)** |  |
| **Mid upper arm circumference** |  |  | 0.09 |  |  | **0.05** |
| <20 | 43 (3.4%) | 8 (5.4%) |  | **37 (3.3%)** | **14 (5.2%)** |  |
| ≥20-<23 | 613 (49.0%) | 82 (55.8%) |  | **550 (48.7%)** | **145 (54.1%)** |  |
| ≥23 | 595 (47.6%) | 57 (38.8%) |  | **543 (48.1%)** | **109 (40.7%)** |  |
| **Parity** |  |  | 0.87 |  |  | 0.14 |
| 0 | 582 (45.0%) | 68 (45.3%) |  | 532 (45.6%) | 118 (42.9%) |  |
| 1-2 | 496 (38.4%) | 55 (36.7%) |  | 451 (38.6%) | 100 (36.4%) |  |
| 3+ | 214 (16.6%) | 27 (18.0%) |  | 184 (15.8%) | 57 (20.7%) |  |
| **Weeks postpartum at Vaginal sample collection, mean (SD)^1^** | 13.2 (2.1) | 13.1 (1.8) | 0.66 | 13.2 (2.1) | 13.3 (2.1) | 0.50 |
| **Gestational age at outcome (weeks)^2^** | 32.6 (11.9) | 33.8 (10.4) | 0.25 | **32.4 (12.0)** | **34.1 (10.4)** | **0.037** |
| **Weeks since previous pregnancy** | **255.6 (144.7)** | **219.7 (123.8)** | **0.03** | 254.5 (144.4) | 241.5 (137.0) | 0.31 |
| **Months since previous pregnancy (category)** |  |  | 0.80 |  |  | 0.64 |
| <18 months | 47 (6.4%) | 6 (7.1%) |  | 44 (6.7%) | 9 (5.7%) |  |
| ≥ 18 months | 686 (93.6%) | 78 (92.9%) |  | 614 (93.3%) | 150 (94.3%) |  |
| **Age at first marriage (yrs)** |  |  | 0.49 |  |  | 0.32 |
| <15 | 544 (59.0%) | 53 (64.6%) |  | 497 (58.5%) | 100 (64.9%) |  |
| 15-18 | 258 (28.0%) | 18 (22.0%) |  | 240 (28.2%) | 36 (23.4%) |  |
| 18+ | 120 (13.0%) | 11 (13.4%) |  | 113 (13.3%) | 18 (11.7%) |  |
| **Outcome of previous pregnancy** |  |  | 0.15 |  |  | 0.10 |
| At least one live birth | 626 (84.6%) | 76 (90.5%) |  | 560 (84.2%) | 142 (89.3%) |  |
| Stillbirth/miscarriage | 114 (15.4%) | 8 (9.5%) |  | 105 (15.8%) | 17 (10.7%) |  |
| **Pregnancy outcome^3^** |  |  | 0.18 |  |  | **0.035** |
| Term birth | 792 (61.2%) | 93 (62.0%) |  | **713 (61.0%)** | **172 (62.5%)** |  |
| Preterm birth | 206 (15.9%) | 33 (22.0%) |  | **181 (15.5%)** | **58 (21.1%)** |  |
| Miscarriage | 107 (8.3%) | 8 (5.3%) |  | **102 (8.7%)** | **13 (4.7%)** |  |
| Stillbirth | 37 (2.9%) | 2 (1.3%) |  | **33 (2.8%)** | **6 (2.2%)** |  |
| Abortion | 152 (11.7%) | 14 (9.3%) |  | **140 (12.0%)** | **26 (9.5%)** |  |
| **Breast feeding status^4^** |  |  | 0.60 |  |  | 0.73 |
| No/partial breastfeeding | 814 (87.9%) | 100 (86.2%) |  | 733 (87.9%) | 181 (87.0%) |  |
| Exclusive breastfeeding | 112 (12.1%) | 16 (13.8%) |  | 101 (12.1%) | 27 (13.0%) |  |
| **SES characteristics** | | | |  |  |  |
| **Women’s education** |  |  | **0.05** |  |  | 0.19 |
| No schooling | **476 (36.8%)** | **64 (42.7%)** |  | 428 (36.7%) | 112 (40.7%) |  |
| Class 1-7 | **442 (34.2%)** | **57 (38.0%)** |  | 401 (34.4%) | 98 (35.6%) |  |
| Class 8-14 | **374 (28.9%)** | **29 (19.3%)** |  | 338 (29.0%) | 65 (23.6%) |  |
| **Husband’s education** |  |  | 0.09 |  |  | 0.12 |
| No schooling | 572 (46.7%) | 80 (56.3%) |  | 513 (46.4%) | 139 (53.3%) |  |
| Class 1-7 | 300 (24.5%) | 30 (21.1%) |  | 271 (24.5%) | 59 (22.6%) |  |
| Class 8-14 | 353 (28.8%) | 32 (22.5%) |  | 322 (29.1%) | 63 (24.1%) |  |
| **Living standard index** |  |  | 0.06 |  |  | 0.11 |
| Lowest | 396 (30.6%) | 55 (36.7%) |  | 270 (23.1%) | 82 (29.8%) |  |
| Middle | 429 (33.2%) | 55 (36.7%) |  | 292 (25.0%) | 68 (24.7%) |  |
| High | 468 (36.2%) | 40 (26.7%) |  | 298 (25.5%) | 59 (21.5%) |  |
| **Women literacy** | **651 (50.3%)** | **61 (40.7%)** | **0.03** | 588 (50.3%) | 124 (45.1%) | 0.12 |
| **Husband literacy** | 637 (49.6%) | 63 (42.3%) | 0.09 | 577 (49.8%) | 123 (44.9%) | 0.15 |
| **Religion** |  |  | 0.95 |  |  | 0.21 |
| Muslim | 1,205 (93.2%) | 140 (93.3%) |  | 1,084 (92.8%) | 261 (94.9%) |  |
| Hindu | 88 (6.8%) | 10 (6.7%) |  | 84 (7.2%) | 14 (5.1%) |  |
| **Behavioral characteristics^5^** | | | |  |  |  |
| **Use soap when bathing^6^** |  |  | **0.016** |  |  | **0.002** |
| Never/Sometimes | **270 (27.3%)** | **35 (39.3%)** |  | **242 (26.5%)** | **63 (38.4%)** |  |
| Always | **718 (72.7%)** | **54 (60.7%)** |  | **671 (73.5%)** | **101 (61.6%)** |  |
| **Water source when bathing** |  |  | 0.48 |  |  | 0.63 |
| Not pond/river/lake | 375 (31.4%) | 39 (28.5%) |  | 340 (31.4%) | 74 (29.8%) |  |
| Pond/river/lake | 818 (68.6%) | 98 (71.5%) |  | 742 (68.6%) | 174 (70.2%) |  |
| **Wash vaginal area when bathing** |  |  | 0.47 |  |  | 0.54 |
| No | 150 (15.2%) | 11 (12.4%) |  | 134 (14.7%) | 27 (16.6%) |  |
| Yes | 835 (84.8%) | 78 (87.6%) |  | 777 (85.3%) | 136 (83.4%) |  |
| **Wash vaginal area**  **frequency** |  |  | **0.026** |  |  | 0.16 |
| Occasionally | **232 (27.8%)** | **31 (39.7%)** |  | 217 (28.0%) | 46 (33.8%) |  |
| Every time | **602 (72.2%)** | **47 (60.3%)** |  | 559 (72.0%) | 90 (66.2%) |  |
| **Clean anal area after defecation** |  |  | 0.29 |  |  | 0.26 |
| Front to back | 549 (56.4%) | 54 (60.7%) |  | 502 (55.8%) | 101 (62.3%) |  |
| Back to front | 89 (9.1%) | 11 (12.4%) |  | 85 (9.4%) | 15 (9.3%) |  |
| Either way | 335 (34.4%) | 24 (27.0%) |  | 313 (34.8%) | 46 (28.4%) |  |
| **Entered water up to hips in last 30 days^7^** |  |  | 0.48 |  |  | 0.38 |
| Never up to hips | 1,168 (98.0%) | 132 (97.1%) |  | 1,060 (98.1%) | 240 (97.2%) |  |
| Up to hips | 24 (2.0%) | 4 (2.9%) |  | 21 (1.9%) | 7 (2.8%) |  |
| **Used menstrual cloth prior to pregnancy** |  |  | **0.047** |  |  | 0.076 |
| No | **229 (23.2%)** | **29 (32.6%)** |  | 210 (23.0%) | 48 (29.4%) |  |
| Yes | **758 (76.8%)** | **60 (67.4%)** |  | 703 (77.0%) | 115 (70.6%) |  |
| **Re-used menstrual**  **cloth^8^** |  |  | **0.003** |  |  | 0.19 |
| No | **16 (2.1%)** | **5 (8.3%)** |  | 16 (2.3%) | 5 (4.3%) |  |
| Yes | **742 (97.9%)** | **55 (91.7%)** |  | 687 (97.7%) | 110 (95.7%) |  |
| **Water source to wash**  **menstrual cloth** |  |  | 0.59 |  |  | 0.42 |
| Water only | 4 (0.5%) | 0 (0.0%) |  | 4 (0.6%) | 0 (0.0%) |  |
| Water and  soap/alkali | 738 (99.5%) | 55 (100.0%) |  | 683 (99.4%) | 110 (100.0%) |  |
| **Menstruation resumed after pregnancy outcome** |  |  | 0.46 |  |  | 0.17 |
| No | 573 (61.4%) | 76 (65.0%) |  | 510 (60.8%) | 139 (65.9%) |  |
| Yes | 360 (38.6%) | 41 (35.0%) |  | 329 (39.2%) | 72 (34.1%) |  |
| **Family planning use after pregnancy** |  |  | **0.030** |  |  | 0.19 |
| No FP | **924 (77.5%)** | **114 (83.2%)** |  | 838 (77.4%) | 200 (80.6%) |  |
| Oral pills | **215 (18.0%)** | **19 (13.9%)** |  | 193 (17.8%) | 41 (16.5%) |  |
| IUD | **2 (0.2%)** | **0 (0.0%)** |  | 2 (0.2%) | 0 (0.0%) |  |
| Depo-provera injection | **23 (1.9%)** | **2 (1.5%)** |  | 22 (2.0%) | 3 (1.2%) |  |
| Condoms | **29 (2.4%)** | **1 (0.7%)** |  | 27 (2.5%) | 3 (1.2%) |  |
| Other | **0 (0.0%)** | **1 (0.7%)** |  | 0 (0.0%) | 1 (0.4%) |  |
| **Self-reported BV treatment^9^** |  |  | 0.96 |  |  | 0.85 |
| Oral tablet/syrup | 79 (79.8%) | 3 (75.0%) |  | 72 (80.9%) | 10 (71.4%) |  |
| Intravaginal tablet | 1 (1.0%) | 0 (0.0%) |  | 1 (1.1%) | 0 (0.0%) |  |
| Intravaginal cream or  ointment | 1 (1.0%) | 0 (0.0%) |  | 1 (1.1%) | 0 (0.0%) |  |
| Sinkara syrup | 1 (1.0%) | 0 (0.0%) |  | 1 (1.1%) | 0 (0.0%) |  |
| Leaves or some herbal  preparation | 11 (11.1%) | 1 (25.0%) |  | 9 (10.1%) | 3 (21.4%) |  |
| Other | 6 (6.1%) | 0 (0.0%) |  | 5 (5.6%) | 1 (7.1%) |  |
| **Antibiotic treatment for BV (early or late pregnancy)^10^** |  |  | **<0.001** |  |  | **<0.001** |
| No treatment/no BV | **1,204 (93.0%)** | **116 (77.3%)** |  | **1,087 (93.0%)** | **233 (84.4%)** |  |
| BV/no treatment | **51 (3.9%)** | **21 (14.0%)** |  | **49 (4.2%)** | **23 (8.3%)** |  |
| BV/treatment | **40 (3.1%)** | **13 (8.7%)** |  | **33 (2.8%)** | **20 (7.2%)** |  |
| **Chewed betelnut** |  |  | 0.43 |  |  | 0.74 |
| No | 395 (31.8%) | 42 (28.6%) |  | 355 (31.6%) | 82 (30.6%) |  |
| Yes | 848 (68.2%) | 105 (71.4%) |  | 767 (68.4%) | 186 (69.4%) |  |

^1^n=6/1,165 (0.36%) women were met at <77 days at the postpartum visit.

^2^Even though gestational age at outcome (weeks) was significantly associated with Nugent-BV 4-10 postpartum, it was excluded from the final regression models because it did not change the associations in the sensitivity analyses.

^3^Even though pregnancy outcome was significantly associated with Nugent-BV 4-10 postpartum, it was excluded from the final regression models because it did not change the associations in the original model in the sensitivity analyses. Furthermore, while mode of delivery may influence the postpartum microbiota, only 20 women (1.56%) reported having a cesarean section, and no significant association was found between cesarean delivery and postpartum Nugent-BV 7–10 or 4–10.

^4^n=5/1,191 (0.42%) women with live births reported no breastfeeding at 3 months postpartum.

^5^No women reported consuming alcohol; <10% reported any tobacco use at enrollment; >94% of women reported to have resumed sexual intercourse since the end of their pregnancy.

^6^n=5 (0.35%) women reported never using soap when bathing in early pregnancy.

^7^Entering water up to hips for fishing, washing cows, crossing water, or for any other reason in the last 30 days postpartum was not associated with Nugent-BV 7-10 or Nugent-BV 4-10 postpartum.

^8^Even though some variables related to using menstrual cloth prior to pregnancy were significantly associated with Nugent-BV 7-10 or Nugent-BV 4-10 in early pregnancy, they were excluded from sensitivity analyses and the final regression models given that ~98% reported re-using menstrual cloth in early pregnancy, and ~99% women reported using water and soap/alkali to wash menstrual cloth if they reused it. Furthermore, it is less clear how relevant these indicators may be, provided the data were collected in early pregnancy and menses did not occur during pregnancy and could have possibly changed postpartum where a substantial proportion of women reported not having resumed menses.

^9^Type of BV treatment method was asked about postpartum and refers to any treatment the woman received for current abnormal vaginal discharge, not treatment received from the study.

^10^Even though antibiotic treatment for BV during early or late pregnancy was significantly associated with Nugent-BV 7-10 or Nugent-BV 4-10 postpartum, it was excluded from the final regression models because the proportion of women who had symptomatic BV and received treatment was low which limited the robustness of the regression model.

Bold: p<0.1
