# Supplementary material for: Factors associated with Nugent-bacterial vaginosis in pregnancy and postpartum among women in rural northwestern Bangladesh
Source: PLOS Glob Public Health. 2025 Jun 13;5(6):e0004768. doi: 10.1371/journal.pgph.0004768 (PMC12165353; doi:10.1371/journal.pgph.0004768)
Supplement: S7 Table — (DOC) [file pgph.0004768.s008.doc]

**S7 Table. Characteristics of pregnant women by Nugent-BV 4-6 3-months postpartum**

|  | **Nugent score 0-3**  **(n=1,169)** | **Nugent-BV 4-6**  **(n=126)** | **p-value** |
| --- | --- | --- | --- |
| **Treatment Arm** |  |  | 0.65 |
| Placebo | 362 (31.0%) | 35 (27.8%) |  |
| Vitamin A | 379 (32.4%) | 40 (31.7%) |  |
| Beta carotene | 428 (36.6%) | 51 (40.5%) |  |
| **Treatment adherence (0-100) during study** |  |  | 0.56 |
| <95 | 486 (41.6%) | 49 (38.9%) |  |
| ≥ 95 | 683 (58.4%) | 77 (61.1%) |  |
| **Clinical characteristics** | | | |
| **Age at pregnancy ascertainment (y), mean (SD)** | **22.0 (6.2)** | **23.4 (6.9)** | **0.02** |
| **Age (yrs)** |  |  | 0.11 |
| <18 | 300 (25.7%) | 27 (21.4%) |  |
| 18-29 | 712 (61.0%) | 74 (58.7%) |  |
| ≥30 | 156 (13.4%) | 25 (19.8%) |  |
| **BMI (category)** |  |  | 0.56 |
| Underweight | 477 (41.2%) | 58 (46.0%) |  |
| Normal weight | 646 (55.8%) | 64 (50.8%) |  |
| Overweight or obese | 34 (2.9%) | 4 (3.2%) |  |
| **BMI (category 2)** |  |  | 0.30 |
| Normal BMI | 680 (58.8%) | 68 (54.0%) |  |
| Low BMI (<18.5) | 477 (41.2%) | 58 (46.0%) |  |
| **Mid upper arm circumference** |  |  | 0.42 |
| <20 | 37 (3.3%) | 6 (5.0%) |  |
| ≥20-<23 | 550 (48.7%) | 63 (52.1%) |  |
| ≥23 | 543 (48.1%) | 52 (43.0%) |  |
| **Parity** |  |  | 0.06 |
| 0 | 532 (45.6%) | 50 (40.0%) |  |
| 1-2 | 451 (38.6%) | 45 (36.0%) |  |
| 3+ | 184 (15.8%) | 30 (24.0%) |  |
| **Weeks postpartum at Vaginal sample collection, mean (SD)^1^** | 13.2 (2.1) | 13.4 (2.4) | 0.18 |
| **Gestational age at outcome (weeks)^2^** | 32.4 (12.0) | 34.4 (10.4) | 0.077 |
| **Weeks since previous pregnancy** | 244.5 (144.9) | 257.9 (151.3) | 0.47 |
| **Months since previous pregnancy (category)** |  |  | 0.85 |
| <18 months | 50 (7.9%) | 5 (7.2%) |  |
| ≥ 18 months | 583 (92.1%) | 64 (92.8%) |  |
| **Age at first marriage (yrs)** |  |  | 0.49 |
| <15 | 497 (58.5%) | 47 (65.3%) |  |
| 15-18 | 240 (28.2%) | 18 (25.0%) |  |
| 18+ | 113 (13.3%) | 7 (9.7%) |  |
| **Outcome of previous pregnancy** |  |  | 0.39 |
| At least one live birth | 560 (84.2%) | 66 (88.0%) |  |
| Stillbirth/miscarriage | 105 (15.8%) | 9 (12.0%) |  |
| **Pregnancy outcome**^3^ |  |  | 0.27 |
| Term birth | 713 (61.0%) | 79 (63.2%) |  |
| Preterm birth | 181 (15.5%) | 25 (20.0%) |  |
| Miscarriage | 102 (8.7%) | 5 (4.0%) |  |
| Stillbirth | 33 (2.8%) | 4 (3.2%) |  |
| Abortion | 140 (12.0%) | 12 (9.6%) |  |
| **Breast feeding status^4^** |  |  | 0.60 |
| No/partial breastfeeding | 814 (87.9%) | 100 (86.2%) |  |
| Exclusive breastfeeding | 112 (12.1%) | 16 (13.8%) |  |
| **SES characteristics** | | | |
| **Women’s education** |  |  | 0.19 |
| No schooling | 428 (36.7%) | 112 (40.7%) |  |
| Class 1-7 | 401 (34.4%) | 98 (35.6%) |  |
| Class 8-14 | 338 (29.0%) | 65 (23.6%) |  |
| **Husband’s education** |  |  | 0.12 |
| No schooling | 513 (46.4%) | 139 (53.3%) |  |
| Class 1-7 | 271 (24.5%) | 59 (22.6%) |  |
| Class 8-14 | 322 (29.1%) | 63 (24.1%) |  |
| **Living standard index** |  |  | 0.35 |
| Lowest | 352 (30.1%) | 44 (35.2%) |  |
| Middle | 394 (33.7%) | 35 (28.0%) |  |
| High | 422 (36.1%) | 46 (36.8%) |  |
| **Women literacy** | 588 (50.3%) | 63 (50.4%) | 0.99 |
| **Husband literacy** | 577 (49.8%) | 60 (48.0%) | 0.70 |
| **Religion** |  |  | 0.09 |
| Muslim | 1,084 (92.8%) | 121 (96.8%) |  |
| Hindu | 84 (7.2%) | 4 (3.2%) |  |
| **Behavioral characteristics^5^** | | | |
| **Use soap when bathing^6^** |  |  | **0.043** |
| Never/Sometimes | **242 (26.5%)** | **28 (37.3%)** |  |
| Always | **671 (73.5%)** | **47 (62.7%)** |  |
| **Water source when bathing** |  |  | 0.98 |
| Not pond/river/lake | 340 (31.4%) | 35 (31.5%) |  |
| Pond/river/lake | 742 (68.6%) | 76 (68.5%) |  |
| **Wash vaginal area when bathing** |  |  | 0.11 |
| No | 134 (14.7%) | 16 (21.6%) |  |
| Yes | 777 (85.3%) | 58 (78.4%) |  |
| **Wash vaginal area**  **frequency** |  |  | 0.73 |
| Occasionally | 217 (28.0%) | 15 (25.9%) |  |
| Every time | 559 (72.0%) | 43 (74.1%) |  |
| **Clean anal area after defecation** |  |  | 0.29 |
| Front to back | 502 (55.8%) | 47 (64.4%) |  |
| Back to front | 85 (9.4%) | 4 (5.5%) |  |
| Either way | 313 (34.8%) | 22 (30.1%) |  |
| **Entered water up to hips in last 30 days^7^** |  |  | 0.59 |
| Never up to hips | 1,060 (98.1%) | 108 (97.3%) |  |
| Up to hips | 21 (1.9%) | 3 (2.7%) |  |
| **Used menstrual cloth prior to pregnancy** |  |  | 0.60 |
| No | 210 (23.0%) | 19 (25.7%) |  |
| Yes | 703 (77.0%) | 55 (74.3%) |  |
| **Re-used menstrual**  **cloth** |  |  | 0.26 |
| No | 16 (2.3%) | 0 (0.0%) |  |
| Yes | 687 (97.7%) | 55 (100.0%) |  |
| **Water source to wash**  **menstrual cloth** |  |  | 0.57 |
| Water only | 4 (0.6%) | 0 (0.0%) |  |
| Water and  soap/alkali | 683 (99.4%) | 55 (100.0%) |  |
| **Menstruation resumed after pregnancy outcome** |  |  | 0.24 |
| No | 510 (60.8%) | 63 (67.0%) |  |
| Yes | 329 (39.2%) | 31 (33.0%) |  |
| **Family planning use after pregnancy** |  |  | 0.86 |
| No FP | 838 (77.4%) | 86 (77.5%) |  |
| Oral pills | 193 (17.8%) | 22 (19.8%) |  |
| IUD | 2 (0.2%) | 0 (0.0%) |  |
| Depo-provera injection | 22 (2.0%) | 1 (0.9%) |  |
| Condoms | 27 (2.5%) | 2 (1.8%) |  |
| **Self-reported BV treatment^8^** |  |  | 0.91 |
| Oral tablet/syrup | 72 (80.9%) | 7 (70.0%) |  |
| Intravaginal tablet | 1 (1.1%) | 0 (0.0%) |  |
| Intravaginal cream or  ointment | 1 (1.1%) | 0 (0.0%) |  |
| Sinkara syrup | 1 (1.1%) | 0 (0.0%) |  |
| Leaves or some herbal  preparation | 9 (10.1%) | 2 (20.0%) |  |
| Other | 5 (5.6%) | 1 (10.0%) |  |
| **Antibiotic treatment for BV (early or late pregnancy)^9^** |  |  | 0.095 |
| No treatment/no BV | 1,087 (93.0%) | 117 (92.9%) |  |
| BV/no treatment | 49 (4.2%) | 2 (1.6%) |  |
| BV/treatment | 33 (2.8%) | 7 (5.6%) |  |
| **Chewed betelnut** |  |  | 0.75 |
| No | 355 (31.6%) | 40 (33.1%) |  |
| Yes | 767 (68.4%) | 81 (66.9%) |  |

^1^No women reported consuming alcohol; <10% reported any tobacco use at enrollment.

^2^Even though gestational age at outcome (weeks) was significantly associated with Nugent-BV 4-6 postpartum, it was excluded from the final regression models because it did not change the associations in the original model in the sensitivity analyses.

^3^While mode of delivery may influence the postpartum microbiota, only 20 women (1.56%) reported having a cesarean section, and no significant association was found between cesarean delivery and postpartum Nugent-BV 4-6.

^4^n=5/1,191 (0.42%) women with live births reported no breastfeeding at the 3 months postpartum visit.

^5^No women reported any alcohol consumption, and <10% reported any tobacco use at enrollment. >94% of women reported to have resumed sexual intercourse since the end of their pregnancy.

^6^n=5 (0.35%) women reported never using soap when bathing in early pregnancy.

^7^Entering water up to hips for fishing, washing cows, crossing water, or for any other reason in the last 30 days postpartum was not associated with Nugent-BV 4-6 postpartum.

^8^Type of BV treatment method was asked about postpartum and refers to any treatment the woman received for current abnormal vaginal discharge, not treatment received from the study.

^9^Even though antibiotic treatment for BV during early or late pregnancy was significantly associated with Nugent-BV 7-10 or Nugent-BV 4-10 postpartum, it was excluded from the final regression models the proportion of women who had symptomatic BV and received treatment was low.

Bold: p<0.1
