# Supplementary material for: Factors associated with Nugent-bacterial vaginosis in pregnancy and postpartum among women in rural northwestern Bangladesh
Source: PLOS Glob Public Health. 2025 Jun 13;5(6):e0004768. doi: 10.1371/journal.pgph.0004768 (PMC12165353; doi:10.1371/journal.pgph.0004768)
Supplement: S8 Table — (DOC) [file pgph.0004768.s009.doc]

S8 Table. Adjusted associations between Nugent-BV 4-6 and potential factors in early and late pregnancy and 3-months postpartum

| **Variables** | **Early pregnancy (n=1,249)** | **Late pregnancy (n=913)** | **Postpartum (n=1,278)** |
| --- | --- | --- | --- |
| **Age** |  |  |  |
| <18 | Ref | Ref | Ref |
| 18-29 | 1.45 (0.68, 3.08) | 0.86 (0.55, 1.36) | 1.19 (0.76, 1.86) |
| ≥30 | **2.87 (1.04, 7.90)** | 0.99 (0.24, 4.05) | 1.44 (0.72, 2.90) |
| **BMI** |  |  |  |
| Normal BMI | Ref | Ref | Ref |
| Low BMI (<18.5) | 1.02 (0.69, 1.49) | **0.51 (0.34, 0.79)*** | 1.17 (0.77, 1.79) |
| **GA at vaginal sample collection (EP/LP) / weeks since delivery (PP)^2^** | 0.98 (0.93, 1.02) | 1.17 (0.97, 1.41) | 1.05 (0.98, 1.11) |
| **Wealth (LSI)^3^** |  |  |  |
| Lowest | Ref | Ref | Ref |
| Middle | 0.70 (0.41, 1.21) | 1.04 (0.58, 1.86) | 0.76 (0.45, 1.29) |
| High | 0.79 (0.38, 1.65) | 0.56 (0.21, 1.45) | 0.89 (0.48, 1.68) |
| **Maternal education** |  |  |  |
| No education | Ref | Ref | Ref |
| Class 1-7 | 0.91 (0.50, 1.63) | 0.89 (0.52, 1.52) | 1.08 (0.73, 1.60) |
| Class 8-14 | 0.73 (0.38, 1.42) | 0.62 (0.25, 1.55) | 1.21 (0.64, 2.29) |
| **Religion** |  |  |  |
| Muslim | Ref | Ref | Ref |
| Hindu | 0.94 (0.34, 2.59) | 1.28 (0.57, 2.89) | 0.46 (0.17, 1.27) |
| **Parity** |  |  |  |
| 0 | Ref | Ref | Ref |
| 1-2 | 0.71 (0.38, 1.34) | 0.62 (0.45, 1.12) | 0.99 (0.57, 1.70) |
| 3+ | 0.90 (0.43, 1.86) | 0.66 (0.23, 1.85) | 1.41 (0.79, 2.51) |
| **Use soap when bathing** |  |  |  |
| Never/Sometimes | Ref | - | - |
| Always | 1.08 (0.64, 1.82) | - | - |
| **Antenatal care visits^4^** |  |  |  |
| 0 visits | Ref | - | - |
| At least 1 visit | - | 0.62 (0.36, 1.08) | - |

^1^This table presents adjusted prevalence ratios and associated confidence intervals prior to multiple comparisons correction. Results marked with an asterisk indicate that the p-value for that test remained significant (p<0.05) after Benjamini-Hochberg adjustment or that the p-value was significant and this test did not require multiple comparisons correction because this variable was only used in one final model. Bold tests without an asterisk indicate a test that was significant (p<0.05) prior to Benjamini-Hochberg adjustment but not afterwards.

^2^GA: gestational age; EP: early pregnancy; LP: late pregnancy; PP: postpartum

^3^LSI: living standard index

^4^The antenatal care visit variable was only included in late pregnancy since there was no significant association in early pregnancy or postpartum

Note: the final adjusted regression models used a generalized estimation equation (GEE) with a log link, assuming an exchangeable correlation structure and accounting for the cluster (study sector) to adjust for the cluster randomized study design. The regression models adjusted for trial supplementation group in each analysis.
