# Supplementary material for: Factors associated with Nugent-bacterial vaginosis in pregnancy and postpartum among women in rural northwestern Bangladesh
Source: PLOS Glob Public Health. 2025 Jun 13;5(6):e0004768. doi: 10.1371/journal.pgph.0004768 (PMC12165353; doi:10.1371/journal.pgph.0004768)
Supplement: S9 Table — (DOC) [file pgph.0004768.s010.doc]

**S9 Tables: Sensitivity analyses with additional potential factors associated with Nugent-BV 7-10, Nugent-BV 4-10, and Nugent-BV 4-6**

**S9 Table A1.** Adjusted associations between Nugent-BV 7-10 and Nugent-BV 4-10 and potential factors in early pregnancy (bathing water source)

| **Variables** | **Model 1 (original model)**  **N=1,351** | | **Model 2 (bathing water source)**  **N=1,351** | |
| --- | --- | --- | --- | --- |
|  | Nugent-BV 7-10 | Nugent-BV 4-10 | Nugent-BV 7-10 | Nugent-BV 4-10 |
| **Age** |  |  |  |  |
| <18 | Ref | Ref | Ref | Ref |
| 18-29 | 1.09 (0.60, 1.99) | 1.19 (0.77, 1.84) | 1.17 (0.77, 1.82) | 1.18 (0.77, 1.82) |
| ≥30 | 1.72 (0.79, 3.76) | **2.00 (1.04, 3.86)** | **1.97 (1.03, 3.76)** | **1.97 (1.03, 3.76)** |
| **BMI** |  |  |  |  |
| Normal BMI | Ref | Ref | Ref | Ref |
| Low BMI (<18.5) | 0.94 (0.71, 1.23) | 0.97 (0.78, 1.21) | 0.98 (0.79, 1.22) | 0.98 (0.79, 1.22) |
| **GA at vaginal sample collection (EP/LP) / weeks since delivery (PP)^1^** | 1.00 (0.97, 1.04) | 0.99 (0.96, 1.03) | 0.99 (0.96, 1.02) | 0.99 (0.96, 1.02) |
| **Wealth (LSI)^2^** |  |  |  |  |
| Lowest | Ref | Ref | Ref | Ref |
| Middle | 1.05 (0.69, 1.62) | 0.91 (0.68, 1.23) | 0.89 (0.66, 1.20) | 0.89 (0.66, 1.20) |
| High | 1.05 (0.64, 1.73) | 0.94 (0.65, 1.36) | 0.89 (0.62, 1.28) | 0.89 (0.62, 1.28) |
| **Maternal education** |  |  |  |  |
| No education | Ref | Ref | Ref | Ref |
| Class 1-7 | 0.72 (0.42, 1.23) | 0.81 (0.54, 1.23) | 0.82 (0.54, 1.24) | 0.82 (0.54, 1.24) |
| Class 8-14 | 0.72 (0.41, 1.25) | 0.73 (0.46, 1.18) | 0.71 (0.45, 1.14) | 0.71 (0.45, 1.14) |
| **Religion** |  |  |  |  |
| Muslim | Ref | Ref | Ref | Ref |
| Hindu | 1.65 (0.86, 3.17) | 1.32 (0.74, 2.36) | 1.24 (0.67, 2.27) | 1.24 (0.67, 2.27) |
| **Parity** |  |  |  |  |
| 0 | Ref | Ref | Ref | Ref |
| 1-2 | 0.80 (0.43, 1.48) | 0.77 (0.55, 1.07) | 0.78 (0.56, 1.08) | 0.78 (0.56, 1.08) |
| 3+ | 0.77 (0.39, 1.52) | 0.80 (0.49, 1.30) | 0.82 (0.52, 1.32) | 0.82 (0.52, 1.32) |
| **Use soap when bathing** |  |  |  |  |
| Never/Sometimes | Ref | Ref | Ref | Ref |
| Always | **0.64 (0.43, 0.96)** | 0.77 (0.59, 1.02) | 0.77 (0.59, 1.02) | 0.77 (0.59, 1.02) |
| **Wash birth canal when bathing (early pregnancy)** |  |  |  |  |
| Not pond/river/lake | - | - | Ref | Ref |
| Pond/river/lake | - | - | 0.73 (0.50, 1.06) | 0.73 (0.50, 1.06) |

^1^GA: gestational age; EP: early pregnancy; LP: late pregnancy; PP: postpartum

^2^LSI: living standard index

Bold: p<0.05

Note: the final adjusted regression models used a generalized estimation equation (GEE) with a log link, assuming an exchangeable correlation structure and accounting for the cluster (study sector) to adjust for the cluster randomized study design.

**S9 Table B1. Adjusted associations between Nugent-BV 7-10 and Nugent-BV 4-10 and potential factors in late pregnancy (months since last pregnancy, age at first marriage)**

| **Variables** | **Model 1 (original model)**  **N=970** | | **Model 2 (months since last pregnancy)**  **N=483** | | **Model 3 (age at first marriage)**  **N=762** | |
| --- | --- | --- | --- | --- | --- | --- |
|  | Nugent-BV 7-10 | Nugent-BV 4-10 | Nugent-BV 7-10 | Nugent-BV 4-10 | Nugent-BV 7-10 | Nugent-BV 4-10 |
| **Age** |  |  |  |  |  |  |
| <18 | Ref | Ref | Ref | Ref | Ref | Ref |
| 18-29 | 0.72 (0.38, 1.35) | 0.80 (0.57, 1.11) | 0.56 (0.12, 2.56) | 0.90 (0.22, 3.68) | 0.50 (0.21, 1.19) | 0.64 (0.36, 1.15) |
| ≥30 | 0.74 (0.18, 3.08) | 0.85 (0.37, 1.97) | 0.62 (0.07, 5.11) | 0.90 (0.20, 4.11) | 0.64 (0.10, 4.13) | 0.82 (0.26, 2.56) |
| **BMI** |  |  |  |  |  |  |
| Normal BMI | Ref | Ref | Ref | Ref | Ref | Ref |
| Low BMI (<18.5) | 0.74 (0.45, 1.22) | **0.62 (0.44, 0.87)** | 0.81 (0.39, 1.71) | 0.81 (0.48, 1.38) | 0.56 (0.27, 1.13) | 0.64 (0.40, 1.03) |
| **GA at vaginal sample collection (EP/LP) / weeks since delivery (PP)^1^** | **1.18 (1.04, 1.35)** | **1.17 (1.05, 1.30)** | **1.25 (1.00, 1.55)** | 1.23 (1.05, 1.43) | 1.05 (0.88, 1.27) | 1.20 (0.99, 1.44) |
| **Wealth (LSI)^2^** |  |  |  |  |  |  |
| Lowest | Ref | Ref | Ref | Ref | Ref | Ref |
| Middle | 0.84 (0.41, 1.69) | 0.93 (0.62, 1.41) | 0.77 (0.35, 1.70) | 1.03 (0.60, 1.78) | 1.02 (0.47, 2.20) | 0.97 (0.57, 1.67) |
| High | 0.51 (0.24, 1.08) | 0.55 (0.32, 0.94) | 0.44 (0.11, 1.82) | 0.60 (0.25, 1.46) | 0.55 (0.20, 1.57) | 0.62 (0.34, 1.15) |
| **Maternal education** |  |  |  |  |  |  |
| No education | Ref | Ref | Ref | Ref | Ref | Ref |
| Class 1-7 | 0.81 (0.44, 1.52) | 0.88 (0.61, 1.26) | 0.97 (0.38, 2.50) | 0.77 (0.50, 1.19) | 0.58 (0.24, 1.38) | 0.70 (0.41, 1.19) |
| Class 8-14 | 1.10 (0.51, 2.39) | 0.81 (0.47, 1.40) | 1.01 (0.23, 4.40) | 0.72 (0.28, 1.84) | 1.21 (0.44, 3.31) | 0.92 (0.45, 1.87) |
| **Religion** |  |  |  |  |  |  |
| Muslim | Ref | Ref | Ref | Ref | **Ref** | Ref |
| Hindu | **2.68 (1.52, 4.72)** | **1.88 (1.28, 2.77)** | 1.54 (0.58, 4.11) | 1.82 (0.97, 3.41) | **2.65 (1.43, 4.90)** | 1.73 (0.86, 3.49) |
| **Parity** |  |  |  |  |  |  |
| 0 | Ref | Ref | Ref | Ref | Ref | Ref |
| 1-2 | 1.04 (0.45, 2.42) | 0.80 (0.52, 1.24) | 1.23 (0.19, 8.07) | 1.09 (0.24, 5.00) | 1.23 (0.42, 3.57) | 0.80 (0.39, 1.67) |
| 3+ | 0.73 (0.20, 2.62) | 0.72 (0.39, 1.33) | 0.76 (0.07, 8.41) | 0.92 (0.17, 5.00) | 0.85 (0.16, 4.49) | 0.64 (0.28, 1.48) |
| **Antenatal care visits** |  |  |  |  |  |  |
| 0 visits | Ref | Ref | Ref | Ref | Ref | **Ref** |
| At least 1 visit | 0.53 (0.26, 1.09) | **0.59 (0.38, 0.91)** | 0.93 (0.37, 2.31) | 0.83 (0.44, 1.59) | 0.53 (0.27, 1.08) | **0.50 (0.30, 0.83)** |
| **Months since last pregnancy (enrollment)** |  |  |  |  |  |  |
| <18 months | - | - | Ref | Ref | - | - |
| >= 18 months | - | - | **0.34 (0.14, 0.81)** | 0.80 (0.33, 1.92) | - | - |
| **Age at first marriage (yrs)** |  |  |  |  | - | - |
| <15 | - | - | - | - | Ref | Ref |
| 15-18 | - | - | - | - | 0.49 (0.19, 1.30) | 0.87 (0.50, 1.50) |
| 18+ | - | - | - | - | 0.43 (0.10, 1.94) | 0.67 (0.33, 1.36) |

^1^GA: gestational age; EP: early pregnancy; LP: late pregnancy; PP: postpartum

^2^LSI: living standard index

Bold: p<0.05

Note: the final adjusted regression models used a generalized estimation equation (GEE) with a log link, assuming an exchangeable correlation structure and accounting for the cluster (study sector) to adjust for the cluster randomized study design. The regression models adjusted for trial supplementation group in each analysis.

**S9 Table B2.** Adjusted associations between Nugent-BV 7-10 and Nugent-BV 4-10 and potential factors in late pregnancy (wash birth canal when bathing, antibiotic treatment for BV)

| **Variables** | **Model 1 (original model)**  **N=815** | | **Model 4: wash birth canal when bathing**  **N=812** | | **Model 5: antibiotic treatment in early pregnancy***  **N=970** | |
| --- | --- | --- | --- | --- | --- | --- |
|  | Nugent-BV 7-10 | Nugent-BV 4-10 | Nugent-BV 7-10 | Nugent-BV 4-10 | Nugent-BV 7-10 | Nugent-BV 4-10 |
| **Age** |  |  |  |  |  |  |
| <18 | Ref | Ref | Ref | Ref | Ref | Ref |
| 18-29 | 0.72 (0.38, 1.35) | 0.80 (0.57, 1.11) | 0.45 (0.19, 1.04) | 0.67 (0.40, 1.13) | 0.83 (0.47, 1.46) | 0.86 (0.62, 1.19) |
| ≥30 | 0.74 (0.18, 3.08) | 0.85 (0.37, 1.97) | 0.46 (0.09, 2.45) | 0.95 (0.38, 2.38) | 0.70 (0.19, 2.53) | 0.87 (036, 2.08) |
| **BMI** |  |  |  |  |  |  |
| Normal BMI | Ref | Ref | Ref | **Ref** | Ref | Ref |
| Low BMI (<18.5) | 0.74 (0.45, 1.22) | **0.62 (0.44, 0.87)** | 0.57 (0.28, 1.18) | **0.61 (0.39, 0.93)** | 0.70 (0.43, 1.15) | **0.60 (0.42, 0.85)** |
| **GA at vaginal sample collection (EP/LP) / weeks since delivery (PP)^1^** | **1.18 (1.04, 1.35)** | **1.17 (1.05, 1.30)** | 1.09 (0.91, 1.31) | 1.19 (0.99, 1.41) | 1.23 (1.08, 1.41) | **1.19 (1.05, 1.36)** |
| **Wealth (LSI)^2^** |  |  |  |  |  |  |
| Lowest | Ref | Ref | Ref | Ref | Ref | Ref |
| Middle | 0.84 (0.41, 1.69) | 0.93 (0.62, 1.41) | 0.99 (0.46, 2.15) | 1.06 (0.64, 1.75) | 0.76 (0.37, 1.53) | 0.93 (0.60, 1.44) |
| High | 0.51 (0.24, 1.08) | 0.55 (0.32, 0.94) | 0.52 (0.18, 1.54) | 0.62 (0.32, 1.22) | 0.52 (0.25, 1.06) | 0.57 (0.33, 0.97) |
| **Maternal education** |  |  |  |  |  |  |
| No education | Ref | Ref | Ref | Ref | Ref | Ref |
| Class 1-7 | 0.81 (0.44, 1.52) | 0.88 (0.61, 1.26) | 0.61 (0.24, 1.53) | 0.74 (0.45, 1.24) | 0.94 (0.49, 1.82) | 0.94 (0.65, 1.37) |
| Class 8-14 | 1.10 (0.51, 2.39) | 0.81 (0.47, 1.40) | 1.09 (0.42, 2.80) | 0.82 (0.41, 1.66) | 1.17 (0.54, 2.53) | 0.84 (0.49, 1.42) |
| **Religion** |  |  |  |  |  |  |
| Muslim | Ref | Ref | Ref | Ref | Ref | Ref |
| Hindu | **2.68 (1.52, 4.72)** | **1.88 (1.28, 2.77)** | **2.24 (1.33, 3.76)** | 1.54 (0.80, 2.97) | **2.22 (1.22, 4.05)** | 1.56 (0.99, 2.47) |
| **Parity** |  |  |  |  |  |  |
| 0 | Ref | Ref | Ref | Ref | Ref | Ref |
| 1-2 | 1.04 (0.45, 2.42) | 0.80 (0.52, 1.24) | 1.61 (0.53, 4.88) | 0.80 (0.42, 1.54) | 0.99 (0.47, 2.10) | 0.78 (0.50, 1.23) |
| 3+ | 0.73 (0.20, 2.62) | 0.72 (0.39, 1.33) | 1.25 (0.28, 5.63) | 0.61 (0.28, 1.33) | 0.73 (0.21, 2.53) | 0.69 (0.35, 1.37) |
| **Antenatal care visits** |  |  |  |  |  |  |
| 0 visits | Ref | Ref | Ref | **Ref** | Ref | Ref |
| At least 1 visit | 0.53 (0.26, 1.09) | **0.59 (0.38, 0.91)** | 0.54 (0.27, 1.09) | **0.51 (0.31, 0.83)** | 0.52 (0.26, 1.03) | 0.58 (0.38, 0.89) |
| **Wash birth canal when bathing (early)** |  |  |  |  |  |  |
| No | - | - | Ref | Ref | - | - |
| Yes | - | - | 0.57 (0.26, 1.23) | 1.10 (0.66, 1.81) | - | - |
| **Antibiotic treatment (early)** |  |  |  |  |  |  |
| No BV, no treatment | - | - | - | - | Ref | Ref |
| BV, no treatment | - | - | - | - | **7.12 (4.07, 12.45)** | **4.76 (3.31, 6.86)** |
| BV, treatment | - | - | - | - | 2.15 (0.56, 8.21) | 1.93 (0.75, 4.95) |

^1^GA: gestational age; EP: early pregnancy; LP: late pregnancy; PP: postpartum

^2^LSI: living standard index

Bold: p<0.05

*Model 5 (sensitivity analysis with antibiotic treatment in early pregnancy) ran a multivariable regression model using fam(Poisson) due to non-convergence with fam(bin).

Note: the final adjusted regression models used a generalized estimation equation (GEE) with a log link, assuming an exchangeable correlation structure and accounting for the cluster (study sector) to adjust for the cluster randomized study design. The regression models adjusted for trial supplementation group in each analysis.

**S9 Table C1. Adjusted associations between Nugent-BV 7-10 and Nugent-BV 4-10 and potential factors 3-months** postpartum (resumed menstruation, months since last pregnancy, antibiotic treatment for Nugent-BV 7-10)

| **Variables** | **Model 1: original model**  **N=1,428** | | **Model 2: resumed menstruation (postpartum)**  **N=1,040** | | **Model 3: Months since last pregnancy (enrollment)**  **N=775** | | **Model 4: Antibiotic treatment in early or late pregnancy***  **N=1,428** | |
| --- | --- | --- | --- | --- | --- | --- | --- | --- |
|  | Nugent-BV 7-10 | Nugent-BV 4-10 | Nugent-BV 7-10 | Nugent-BV 4-10 | Nugent-BV 7-10 | Nugent-BV 4-10 | Nugent-BV 7-10 | Nugent-BV 4-10 |
| **Age** |  |  |  |  |  |  |  |  |
| <18 | Ref | Ref | Ref | Ref | Ref | Ref | Ref | Ref |
| 18-29 | 0.66 (0.42, 1.04) | 0.87 (0.64, 1.18) | **0.59 (0.35, 0.99)** | 0.92 (0.64, 1.31) | 0**.37 (0.14, 0.98)** | 0.57 (0.28, 1.16) | 0.67 (0.44, 1.04) | 0.87 (0.65, 1.16) |
| ≥30 | 0.94 (0.50, 1.79) | 1.14 (0.71, 1.83) | 0.80 (0.36, 1.76) | 1.20 (0.66, 2.16) | 0.57 (0.20, 1.68) | 0.82 (0.38, 1.81) | 1.02 (0.54, 1.93) | 1.16 (0.73, 1.85) |
| **BMI** |  |  |  |  |  |  |  |  |
| Normal BMI | Ref | Ref | Ref | Ref | Ref | Ref | Ref | Ref |
| Low BMI (<18.5) | 1.16 (0.82, 1.63) | 1.02 (0.98, 1.06) | 1.26 (0.87, 1.82) | 1.22 (0.93, 1.59) | 1.32 (0.87, 2.01) | 1.11 (0.78, 1.57) | 1.24 (0.89, 1.72) | 1.17 (0.90, 1.53) |
| **GA at vaginal sample collection (EP/LP) / weeks since delivery (PP)^1^** | 0.98 (0.94, 1.03) | 1.16 (0.89, 1.50) | 1.04 (0.96, 1.13) | 1.05 (0.99, 1.12) | 0.99 (0.92, 1.06) | 1.01 (0.96, 1.07) | 0.98 (0.93, 1.03) | 1.01 (0.97, 1.06) |
| **Wealth (LSI)^2^** |  |  |  |  |  |  |  |  |
| Lowest | Ref | Ref | Ref | Ref | Ref | Ref | Ref | Ref |
| Middle | 0.96 (0.62, 1.47) | 0.88 (0.62, 1.24) | 0.83 (0.54, 1.27) | 0.83 (0.58, 1.17) | 0.88 (0.53, 1.47) | 0.83 (0.55, 1.27) | 0.93 (0.62, 1.39) | 0.86 (0.62, 1.20) |
| High | 0.74 (0.45, 1.21) | 0.82 (0.56, 1.20) | 0.80 (0.49, 1.31) | 0.91 (0.63, 1.32) | 0.75 (0.40, 1.42) | 0.86 (0.56, 1.34) | 0.73 (0.46, 1.16) | 0.80 (0.56, 1.16) |
| **Maternal education** |  |  |  |  |  |  |  |  |
| No education | Ref | Ref | Ref | Ref | Ref | Ref | Ref | Ref |
| Class 1-7 | 0.97 (0.65, 1.45) | 1.02 (0.79, 1.32) | 1.15 (0.68, 1.95) | 1.09 (0.78, 1.52) | 0.99 (0.63, 1.54) | 1.05 (0.78, 1.40) | 1.07 (0.72, 1.59) | 1.07 (0.83, 1.38) |
| Class 8-14 | 0.72 (0.41, 1.25) | 0.95 (0.66, 1.36) | 0.85 (0.43, 1.69) | 0.95 (0.58, 1.55) | 0.52 (0.19, 1.43) | 0.78 (0.46, 1.31) | 0.78 (0.46, 1.33) | 1.00 (0.70, 1.43) |
| **Religion** |  |  |  |  |  |  |  |  |
| Muslim | Ref | Ref | Ref | Ref | Ref | Ref | Ref | Ref |
| Hindu | 1.09 (0.61, 1.93) | 0.80 (0.46, 1.39) | 1.30 (0.72, 2.37) | 0.84 (0.45, 1.56) | 1.28 (0.60, 2.74) | 0.99 (0.58, 1.70) | 0.99 (0.54, 1.83) | 0.79 (0.46, 1.35) |
| **Parity** |  |  |  |  |  |  |  |  |
| 0 | Ref | Ref | Ref | Ref | Ref | Ref | Ref | Ref |
| 1-2 | 1.01 (0.66, 1.53) | 1.00 (0.74, 1.34) | 1.23 (0.76, 1.99) | 1.01 (0.74, 1.39) | 1.99 (0.52, 7.60) | 3.70 (0.91, 14.99) | 1.04 (0.69, 1.57) | 1.01 (0.76, 1.33) |
| 3+ | 0.94 (0.56, 1.59) | 1.14 (0.81, 1.58) | 1.51 (0.73, 3.13) | 1.25 (0.80, 1.94) | 1.94 (0.48, 7.92) | **4.29 (1.03, 17.80)** | 0.97 (0.59, 1.61) | 1.15 (0.83, 1.59) |
| **Resumed menstruation (postpartum)** |  |  |  |  |  |  |  |  |
| No | - | - | Ref | Ref | - | - | - | - |
| Yes | - | - | 0.90 (0.61, 1.34) | 0.88 (0.68, 1.13) | - | - | - | - |
| **Months since last pregnancy (enrollment)** |  |  |  |  |  |  |  |  |
| <18 months | - | - | - | - | Ref | Ref | - | - |
| >= 18 months | - | - | - | - | 1.02 (0.45, 2.29) | 0.85 (0.48, 1.51) | - | - |
| **Antibiotic treatment (early or late)** |  |  |  |  |  |  |  |  |
| No BV, no treatment | - | - | - | - | - | - | Ref | Ref |
| BV, no treatment | - | - | - | - | - | - | **3.33 (2.29, 4.85)** | **1.93 (1.43, 2.61)** |
| BV, treatment |  |  |  |  |  |  | **2.95 (1.95, 4.46)** | **2.12 (1.49, 3.00)** |

Bold: p<0.05

*Model 4 (sensitivity analysis with antibiotic treatment in early pregnancy) ran a multivariable regression model using fam(poisson) due to non-convergence with fam(bin).

Note: the final adjusted regression models used a generalized estimation equation (GEE) with a log link, assuming an exchangeable correlation structure and accounting for the cluster (study sector) to adjust for the cluster randomized study design. The regression models adjusted for trial supplementation group in each analysis.

**S9 Table C2. Adjusted associations between Nugent-BV 7-10 and Nugent-BV 4-10 and potential factors 3-months postpartum (family planning categories and using soap when bathing)**

| **Variables** | **Model 1: original model**  **N=1,428** | | **Model 5: family planning**  **(category 1)**  **N=1,315** | | **Model 6: family planning**  **(category 2)**  **N=1,315** | | **Model 7: use soap when bathing**  **N=1,071** | |
| --- | --- | --- | --- | --- | --- | --- | --- | --- |
|  | Nugent-BV 7-10 | Nugent-BV 4-10 | Nugent-BV 7-10 | Nugent-BV 4-10 | Nugent-BV 7-10 | Nugent-BV 4-10 | Nugent-BV 7-10 | Nugent-BV 4-10 |
| **Age** |  |  |  |  |  |  |  |  |
| <18 | Ref | Ref | Ref | Ref | Ref | Ref | Ref | Ref |
| 18-29 | 0.66 (0.42, 1.04) | 0.87 (0.64, 1.18) | **0.58 (0.35, 0.96)** | 0.88 (0.63, 1.25) | **0.58 (0.35, 0.97)** | 0.89 (0.63, 1.25) | 0.62 (0.37, 1.04) | 0.82 (0.59, 1.13) |
| ≥30 | 0.94 (0.50, 1.79) | 1.14 (0.71, 1.83) | 0.85 (0.41, 1.75) | 1.15 (0.66, 2.01) | 0.85 (0.41, 1.74) | 1.14 (0.66, 1.98) | 1.88 (0.90, 3.92) | 1.41 (0.81, 2.42) |
| **BMI** |  |  |  |  |  |  |  |  |
| Normal BMI | Ref | Ref | Ref | Ref | Ref | Ref | Ref | Ref |
| Low BMI (<18.5) | 1.16 (0.82, 1.63) | 1.02 (0.98, 1.06) | 1.16 (0.80, 1.67) | 1.16 (0.88, 1.52) | 1.16 (0.80, 1.67) | 1.16 (0.88, 1.52) | 1.16 (0.72, 1.86) | 1.12 (0.82, 1.53) |
| **GA at vaginal sample collection (EP/LP) / weeks since delivery (PP)^1^** | 0.98 (0.94, 1.03) | 1.16 (0.89, 1.50) | 0.98 (0.91, 1.05) | 1.02 (0.96, 1.08) | 0.98 (0.91, 1.05) | 1.02 (0.96, 1.08) | 0.95 (0.88, 1.02) | 0.98 (0.92, 1.05) |
| **Wealth (LSI)^2^** |  |  |  |  |  |  |  |  |
| Lowest | Ref | Ref | Ref | Ref | Ref | Ref | Ref | Ref |
| Middle | 0.96 (0.62, 1.47) | 0.88 (0.62, 1.24) | 0.94 (0.63, 1.40) | 0.89 (0.64, 1.24) | 0.94 (0.63, 1.40) | 0.89 (0.64, 1.24) | 1.09 (0.65, 1.84) | 1.04 (0.70, 1.57) |
| High | 0.74 (0.45, 1.21) | 0.82 (0.56, 1.20) | 0.75 (0.44, 1.26) | 0.86 (0.59, 1.27) | 0.75 (0.44, 1.26) | 0.87 (0.59, 1.27) | 0.61(0.33, 1.14) | 0.89 (0.52, 155) |
| **Maternal education** |  |  |  |  |  |  |  |  |
| No education | Ref | Ref | Ref | Ref | Ref | Ref | Ref | Ref |
| Class 1-7 | 0.97 (0.65, 1.45) | 1.02 (0.79, 1.32) | 1.00 (0.65, 1.55) | 1.04 (0.80, 1.34) | 1.00 (0.65, 1.55) | 1.04 (0.80, 1.35) | 1.09 (0.69, 1.72) | 1.09 (0.82, 1.46) |
| Class 8-14 | 0.72 (0.41, 1.25) | 0.95 (0.66, 1.36) | 0.79 (0.43, 1.44) | 0.93 (0.61, 1.41) | 0.79 (0.43, 1.44) | 0.92 (0.60, 1.41) | 1.15 (0.65, 2.05) | 0.98 (0.62, 1.56) |
| **Religion** |  |  |  |  |  |  |  |  |
| Muslim | Ref | Ref | Ref | Ref | Ref | Ref | Ref | Ref |
| Hindu | 1.09 (0.61, 1.93) | 0.80 (0.46, 1.39) | 1.12 (0.58, 2.20) | 0.84 (0.45, 1.55) | 1.12 (0.57, 2.23) | 0.84 (0.44, 1.59) | 0.72 (0.25, 2.09) | 0.47 (0.21, 1.02) |
| **Parity** |  |  |  |  |  |  |  |  |
| 0 | Ref | Ref | Ref | Ref | Ref | Ref | Ref | Ref |
| 1-2 | 1.01 (0.66, 1.53) | 1.00 (0.74, 1.34) | 1.19 (0.76, 1.89) | 1.02 (0.74, 1.40) | 1.19 (0.76, 1.89) | 1.02 (0.74, 1.40) | 0.70 (0.39, 1.25) | 0.90 (0.61, 1.33) |
| 3+ | 0.94 (0.56, 1.59) | 1.14 (0.81, 1.58) | 1.14 (0.64, 2.04) | 1.11 (0.76, 1.61) | 1.14 (0.64, 2.05) | 1.13 (0.77, 1.65) | 0.61 (0.32, 1.17) | 1.03 (0.65, 1.64) |
| **FP after pregnancy (postpartum, 2)** |  |  |  |  |  |  |  |  |
| No FP/ IUD/Condoms/Other | - | - | Ref | Ref | - | - | - | - |
| OP/Norplant/Depo | - | - | 0.75 (0.50, 1.14) | 0.87 (0.66, 1.14) | - | - | - | - |
| **FP after pregnancy (postpartum, 3)** |  |  |  |  |  |  |  |  |
| No FP/IUD/Condoms/Other | - | - | - | - | Ref | Ref | - | - |
| Oral Pills | - | - | - | - | 0.75 (0.50, 1.13) | 0.90 (0.68, 1.19) | - | - |
| Norplant/Depo-Provera | - | - |  |  | 0.76 (0.20, 2.82) | 0.59 (0.20, 1.70) | - | - |
| **Use soap when bathing** |  |  |  |  |  |  |  |  |
| Never/Sometimes | - | - |  |  |  |  | Ref | **Ref** |
| Always | - | - |  |  |  |  | 0.70 (0.46, 1.07) | **0.71 (0.53, 0.93)** |

^1^GA: gestational age; EP: early pregnancy; LP: late pregnancy; PP: postpartum

^2^LSI: living standard index

Bold: p<0.05

Note: the final adjusted regression models used a generalized estimation equation (GEE) with a log link, assuming an exchangeable correlation structure and accounting for the cluster (study sector) to adjust for the cluster randomized study design. The regression models adjusted for trial supplementation group in each analysis.

**S9 Table C3. Adjusted associations between Nugent-BV 7-10 and Nugent-BV 4-10 and potential factors 3-months** postpartum (gestational age at outcome, pregnancy outcome)

| **Variables** | **Model 1: original model**  **N=1,428** | | **Model 7: gestational age (weeks) at pregnancy outcome**  **N=1,332** | | **Model 8: pregnancy outcome**  **N=1,427** | |
| --- | --- | --- | --- | --- | --- | --- |
|  | Nugent-BV 7-10 | Nugent-BV 4-10 | Nugent-BV 7-10 | Nugent-BV 4-10 | Nugent-BV 7-10 | Nugent-BV 4-10 |
| **Age** |  |  |  |  |  |  |
| <18 | Ref | Ref | Ref | Ref | **Ref** | Ref |
| 18-29 | 0.66 (0.42, 1.04) | 0.87 (0.64, 1.18) | **0.57 (0.37, 0.89)** | 0.82 (0.61, 1.10) | 0.67 (0.42, 1.05) | 0.88 (0.65, 1.19) |
| ≥30 | 0.94 (0.50, 1.79) | 1.14 (0.71, 1.83) | 0.98 (0.52, 1.85) | 1.32 (0.87, 1.99) | 1.03 (0.53, 2.00) | 1.25 (0.77, 2.04) |
| **BMI** |  |  |  |  |  |  |
| Normal BMI | Ref | Ref | Ref | Ref | Ref | Ref |
| Low BMI (<18.5) | 1.16 (0.82, 1.63) | 1.02 (0.98, 1.06) | 1.14 (0.80, 1.62) | 1.11 (0.85, 1.45) | 1.17 (0.82, 1.65) | 1.18 (0.91, 1.53) |
| **GA at vaginal sample collection (EP/LP) / weeks since delivery (PP)^1^** | 0.98 (0.94, 1.03) | 1.16 (0.89, 1.50) | 1.00 (0.95, 1.05) | 1.03 (0.99, 1.08) | 1.00 (0.96, 1.06) | 1.04 (1.00, 1.08) |
| **Wealth (LSI)^2^** |  |  |  |  |  |  |
| Lowest | Ref | Ref | Ref | Ref | Ref | Ref |
| Middle | 0.96 (0.62, 1.47) | 0.88 (0.62, 1.24) | 0.87 (0.58, 1.31) | 0.78 (0.56, 1.08) | 0.97 (0.63, 1.48) | 0.89 (0.63, 1.26) |
| High | 0.74 (0.45, 1.21) | 0.82 (0.56, 1.20) | 0.75 (0.47, 1.21) | 0.77 (0.53, 1.11) | 0.77 (0.47, 1.25) | 0.85 (0.59, 1.24) |
| **Maternal education** |  |  |  |  |  |  |
| No education | Ref | Ref | Ref | Ref | Ref | Ref |
| Class 1-7 | 0.97 (0.65, 1.45) | 1.02 (0.79, 1.32) | 1.00 (0.68, 1.48) | 1.07 (0.84, 1.35) | 1.00 (0.66, 1.50) | 1.04 (0.79, 1.36) |
| Class 8-14 | 0.72 (0.41, 1.25) | 0.95 (0.66, 1.36) | 0.73 (0.42, 1.28) | 0.98 (0.66, 1.45) | 0.73 (0.42, 1.28) | 0.95 (0.64, 1.40) |
| **Religion** |  |  |  |  |  |  |
| Muslim | Ref | Ref | Ref | Ref | Ref | Ref |
| Hindu | 1.09 (0.61, 1.93) | 0.80 (0.46, 1.39) | 1.12 (0.63, 1.99) | 0.84 (0.49, 1.45) | 1.16 (0.66, 2.03) | 0.84 (0.49, 1.45) |
| **Parity** |  |  |  |  |  |  |
| 0 | Ref | Ref | Ref | Ref | Ref | Ref |
| 1-2 | 1.01 (0.66, 1.53) | 1.00 (0.74, 1.34) | 1.13 (0.75, 1.70) | 0.98 (0.75, 1.29) | 1.05 (0.69, 1.59) | 1.03 (0.77, 1.38) |
| 3+ | 0.94 (0.56, 1.59) | 1.14 (0.81, 1.58) | 1.16 (0.70, 1.90) | 1.30 (0.93, 1.81) | 1.06 (0.63, 1.79) | 1.28 (0.91, 1.81) |
| **Gestational age at outcome (weeks)** | - | - | **1.01 (1.00, 1.03)** | **1.02 (1.01, 1.03)** | - | - |
| **Pregnancy outcome** | - | - | - | - |  |  |
| Term birth | - | - | - | - | Ref | Ref |
| Preterm birth | - | - | - | - | 1.22 (0.80, 1.87) | 1.21 (0.91, 1.61) |
| Miscarriage | - | - | - | - | 0.63 (0.29, 1.38) | **0.52 (0.32, 0.85)** |
| Stillbirth | - | - | - | - | 0.42 (0.12, 1.52) | 0.69 (0.35, 1.38) |
| Abortion | - | - | - | - | 0.67 (0.41, 1.12) | 0.63 (0.44, 0.89) |

^1^GA: gestational age; EP: early pregnancy; LP: late pregnancy; PP: postpartum

^2^LSI: living standard index

Bold: p<0.05

Note: the final adjusted regression models used a generalized estimation equation (GEE) with a log link, assuming an exchangeable correlation structure and accounting for the cluster (study sector) to adjust for the cluster randomized study design. The regression models adjusted for trial supplementation group in each analysis.
