# Supplementary material for: Factors associated with Nugent-bacterial vaginosis in pregnancy and postpartum among women in rural northwestern Bangladesh
Source: PLOS Glob Public Health. 2025 Jun 13;5(6):e0004768. doi: 10.1371/journal.pgph.0004768 (PMC12165353; doi:10.1371/journal.pgph.0004768)
Supplement: S10 Table — (DOC) [file pgph.0004768.s011.doc]

**S10 Table. Summary of reasons why variables were excluded from the final regression models or sensitivity analyses**

|  | **Early pregnancy^1^** | | **Late pregnancy^2^** | | **Postpartum^3^** | |
| --- | --- | --- | --- | --- | --- | --- |
| **Variable** | Reason for selection | Reason for exclusion from final model | Reason for selection | Reason for exclusion from final model | Reason for selection | Reason for exclusion from final model |
|  |  |  |  |  |  |  |
| Bathing water source (early pregnancy) | a-priori, p-value close to 0.1 in Nugent-BV 4-10 bivariate analyses (0.15) | Not significantly associated with Nugent-BV 7-10 or Nugent-BV 4-10; did not affect original model | - | - | - | - |
| Use soap when bathing | - | - | - | - | p-value < 0.1 in bivariate analyses for Nugent-BV 7-10 | Limited sample size (-25%); not significantly associated with Nugent-BV 7-10 or Nugent-BV 4-10 |
| Months since last pregnancy (enrollment) | - | - | p<0.1 in Nugent-BV 7-10 bivariate analyses | Limited sample size (-50.2%); did not affect original model | p<0.1 in Nugent-BV 7-10 bivariate analyses | Limited sample size (-45.7%); not significantly associated with Nugent-BV 7-10 or Nugent-BV 4-10 |
| Age at first marriage (enrollment) | **-** | - | p<0.1 in Nugent-BV 7-10 bivariate analyses | Not significantly associated with Nugent-BV 7-10 or Nugent-BV 4-10; did not affect original model | - | - |
| Wash birth canal when bathing (early pregnancy) | **-** | - | p<0.1 in Nugent-BV 7-10 bivariate analyses | Not significantly associated with Nugent-BV 7-10 or Nugent-BV 4-10; did not affect original model | - | - |
| Resumed menstruation (postpartum) | **-** | - | - | - | a-priori | Limited sample size (-27.2%); not significantly associated with Nugent-BV 7-10 or Nugent-BV 4-10; did not affect original model |
| Postpartum family planning | **-** | - | - | - | p<0.1 in Nugent-BV 7-10 bivariate analyses | Not significantly associated with Nugent-BV 7-10 or Nugent-BV 4-10, did not affect original model |
| Antibiotic treatment in early pregnancy | **-** | - | p<0.1 in Nugent-BV 7-10 bivariate analyses | Few women with asymptomatic Nugent-BV 7-10 with treatment in early pregnancy | - | - |
| Antibiotic treatment in early or late pregnancy | **-** | - | - | - | p<0.1 in Nugent-BV 7-10 bivariate analyses | Few with asymptomatic Nugent-BV 7-10 with treatment in early/late pregnancy |
| Gestational age at outcome (weeks) | **-** | - | - | - | p<0.1 in Nugent-BV 7-10 bivariate analyses | Did not affect original model; original model already included weeks since delivery |
| Pregnancy outcome | **-** | - | - | - | p<0.1 in Nugent-BV 7-10 bivariate analyses | Did not affect original model |

^1^Women’s and husband’s literacy were significantly associated (p<0.1) with Nugent-BV 7-10 and Nugent-BV 4-10 outcome in early pregnancy in the bivariate analyses but were excluded from sensitivity analyses because they were collinear with women’s and husband’s education.

^2^Women’s and husband’s literacy were significantly associated (p<0.1) with Nugent-BV 7-10 and Nugent-BV 4-10 outcome in early pregnancy in the bivariate analyses but were excluded from sensitivity analyses because they were collinear with women and husband’s education. Variables related to re-using cloth in early pregnancy was significantly (p<0.1) associated with Nugent-BV 7-10 in bivariate analyses but were excluded from sensitivity analyses because ~98% reported re-using menstrual cloth in early pregnancy, and ~99% women reported using water and soap/alkali to wash menstrual cloth if they reused it. Furthermore, it is less clear how relevant these indicators may be, provided the data were collected in early pregnancy and menses did not occur during pregnancy. All other variables in the bivariate analyses that share a significantly association with Nugent-BV 7-10 or Nugent-BV 4-10 were included in the sensitivity analyses.

^3^Women’s and husband’s literacy were significantly associated (p<0.1) with Nugent-BV 7-10 and Nugent-BV 4-10 outcome in early pregnancy in the bivariate analyses but were excluded from sensitivity analyses because they were collinear with women’s and husband’s education. MUAC was significantly associated (p<0.10) with Nugent-BV 7-10 and Nugent-BV 4-10 outcomes postpartum in the bivariate analyses, but were collinear with BMI, and therefore excluded from sensitivity analyses. Variables related to re-using cloth (early pregnancy) was significantly (p<0.1) associated with Nugent-BV 7-10 in bivariate analyses but were excluded from sensitivity analyses because ~98% reported re-using menstrual cloth in early pregnancy, and ~99% women reported using water and soap/alkali to wash menstrual cloth if they reused it. Furthermore, it is less clear how relevant these indicators may be, provided the data were collected in early pregnancy and menses did not occur during pregnancy and could have possibly changed postpartum where a substantial proportion of women reported not having resumed menses.
